# Automated recognition of Meso-Cenozoic foraminifera from Senegalese sedimentary deposits using convolutional neural networks

**DOI:** 10.7717/peerj.21437

**Published:** 2026-06-26

**Authors:** Malick Thiam, Sonia Chaabane, Thibault de Garidel-Thoron

**Affiliations:** 1 Aix-Marseille Université, CNRS, IRD, INRAE, CEREGE, Aix-en-Provence, France; 2 University Cheikh Anta Diop, FASTEF, Department of Life and Earth Sciences, Dakar, Senegal; 3 Fondation pour la recherche sur la biodiversité (FRB-CESAB), Montpellier, France; 4 Department of Climate Geochemistry, Max Planck Institute for Chemistry, Mainz, Germany

**Keywords:** Convolutional neural network, Automated recognition, Foraminifera, Continental sediment deposits, Biostratigraphy, Meso-cenozoic, Classification

## Abstract

Fossil foraminifera are key proxies for biostratigraphy and paleoenvironmental reconstructions, providing crucial insights into past ocean conditions and climate evolution. However, their identification remains time-consuming and challenging, particularly in taxonomically complex assemblages and in samples affected by diagenetic alteration. Here, we investigate the application of convolutional neural networks (CNNs) for the automated classification of Meso-Cenozoic foraminifera from the West African margin, including planktonic, benthic, and agglutinated taxa. Three training datasets comprising 11,378 images were used to develop CNN models for microfossil detection, genus-level, and species-level classification. Genus-level classification reached a validation accuracy of 72.4%, outperforming species-level identification (50.2% validation accuracy), as it reduced misclassifications from morphological variability and post-mortem degradation in closely related species. The genus-level CNN performed well for genera with distinct morphological features, such as *Muricohedbergella* sp. and *Heterohelix* sp., but struggled with *Subbotina* spp. and *Dicarinella* spp. due to shared morphological traits and diagenetic alterations. For benthic foraminifera, *Nummulites* sp. was easily identified, while *Gavelinella* sp. posed significant challenges. These results highlight the potential of CNNs for high-throughput classification, while also revealing limitations particularly for taxa with high morphological variability or diagenetic changes. The findings have important implications for biostratigraphy, suggesting that CNNs can enhance genus-level classification in biostratigraphic applications. Further advancements such as multi-view imaging and expanded training datasets could enhance the CNN performance. By integrating deep-learning-based classification with expert biostratigraphy, our study contributes to open the way for quantitative and reproducible reconstructions of Meso-Cenozoic stratigraphic constraints.

## Introduction

Foraminifera, ubiquitous marine protozoans with calcareous shells and chambered tests, are key proxies in oceanography and geosciences, especially within the fields of paleoecology, stratigraphy, and evolutionary studies (Bauch, 1994; Fleisher, 1974; Kucera, 2007). Their significance is underscored by their pivotal role in biostratigraphy, where they allow the correlation of sediments across local, regional to global scales (Loeblich & Tappan, 2015; Bolli, Saunders & Perch-Nielsen, 1985), and they also offer key insights for paleoenvironmental reconstructions (Vincent & Berger, 1981; Shackleton, 1987; Kroon & Ganssen, 1989; Grazzini et al., 1995; Sarnthein et al., 2003; Kucera et al., 2005; De Garidel-Thoron et al., 2005). They encode the deep history of sea surface temperatures, salinity, ocean chemistry, atmospheric pCO_2_ levels (Khozyem et al., 2015; Kucera, 2007; Schiebel et al., 2018), and global ice volume (Zachos et al., 2001). Furthermore, beyond their primary roles in paleoceanographic and paleoecological analyses, planktic foraminifera are used as proxies in comprehensive studies concerning the marine carbonate system (Eggins, De Deckker & Marshall, 2003). For both fundamental academic and applied research fields (biostratigraphy, diagenetic record), it is imperative to identify and classify foraminifera at the species level. Given the intricate and labour-intensive nature of manually identifying under the microscope foraminifera species or morphotypes from sediment samples, there is a pressing need for more streamlined and efficient methodologies, especially when considering the lengthy durations required for high-resolution geochemical analyses.

Micropaleontology is witnessing a revolution in foraminifera identification, greatly owed to technological advancements in both high-throughput imaging techniques and in artificial intelligence, especially deep-learning approaches (Carvalho et al., 2020; Bourel et al., 2020; Marques et al., 2025; Yaqoob et al., 2025; Yu et al., 1996; Yu et al., 2024; Zhang et al., 2024). Historically, pioneers have used morphological analyses of shape, particularly through imaging techniques and the application of Fourier series, to precisely characterize planktonic foraminifera species like *Neogloboquadrina pachyderma* and *Globorotalia truncatulinoides* (Belyea & Thunell, 1984; Healy-Williams, 1983; Healy-Williams, 1984). Although neural networks were developed well before the 1990s, their use for automated microfossil identification was pioneered in the late 1990s (Dollfus & Beaufort, 1999). Following this, the emergence of convolutional neural networks (CNNs) has substantially improved identification, offering an alternative to previous methods (Zhong et al., 2017). CNNs have proven exceptionally effective, and despite initial limitations, their evolution has been rapid and now achieve results close to human experts (Serre, 2019).

Lately, the development of CNN-based tools to streamline the recognition of microfossils (Marchant et al., 2020; Tetard et al., 2020), and to automate the sorting of microfossils with a sorting machine (MiSo; Marchant et al., 2020) aims to sort particles physically and classify vast image datasets. Nevertheless, there is a pressing need to address automation gaps in classifying fossils on very long-term scales beyond the Pleistocene, and to address the impact of various preservation states of foraminiferal tests across varied facies (Marchant et al., 2020; Zhong et al., 2017). Artificial intelligence (AI)-driven microfossil research has often prioritised pristine samples, but test preservation varies strongly with sedimentological facies (carbonate, clay, silica, *etc.*), and recent deep-time studies on fossil pollen have begun to tackle non-pristine, heavily altered material using convolutional neural networks (Romero et al., 2020; Adaïmé, Kong & Punyasena, 2024). More broadly, deep-learning approaches are now explicitly addressing preservation effects in other microfossil groups as well, for example through CNNs trained on modern, fossil, intact and damaged pollen grains (Bourel et al., 2020) and on key fossil calcareous nannofossil species (Zhang et al., 2024), with recent reviews emphasizing the role of taphonomic alteration in fossil image analysis pipelines (Yaqoob et al., 2025).

Here we explore the performance of automated approaches based on CNN recognition to identify Meso-Cenozoic planktonic, benthic and agglutinated fossil foraminifera affected by post-mortem shell transformation like dissolution, over-crusts, infillings, damage, and fragmentation collected from a drillhole from the west African margin (17°27′10″W and 12°37′45″N). In the southwestern region of the Senegalo-Mauritanian basin in Casamance, these deposits form complete and continuous stratigraphic series. This area, located in southern Senegal, provides stratigraphic records from upper Albian to upper Eocene (Castelain, 1965; Monciardini, 1966). These groups offer valuable insights into Meso-Cenozoïc biostratigraphy despite undergoing post-mortem shell transformations, thus serving as important indices for constraining the biostratigraphic sequence. The paleogeographic situation of the studied site is of special interest for understanding Lower Cenomanian to Late Paleocene paleoceanography during the Atlantic Ocean margins extension (Latil-Brun & Flicoteaux, 1986; Latil-Brun & Lucazeau, 1988).

## Biostratigraphy Setting

Several studies in Casamance Basin were conducted on offshore (Ly & Kuhnt, 1994) and onshore (Ly, 1985; Thiam, 2014; Thiam & Sarr, 2014) areas to define ages of stratigraphic units based on microfossil biozones using foraminifera and ostracods assemblages. Onshore stratigraphic results highlight a transgressive marine series dated from Middle Jurassic (Bellion & Guiraud, 1984) to Oligo-Miocene (Flicoteaux & Medus, 1980; Ly, 1985). On offshore domain, scarcity of planktonic foraminifera forces researchers to integrate use of benthic foraminifera (Ly & Kuhnt, 1994) or ostracods (Carbonnel, 1986; Thiam, 2014) to define biozones or to refine planktonic biozonation. Biostratigraphic studies using traditional methods in Casamance Maritime offshore (Ly & Kuhnt, 1994) define the early Paleocene section based on the occurrences of *Morozovella pseudobulloides*. The K/T boundary has been pinpointed at 1,416 mbsf, demarcated by the last occurrence datum (LOD) of the genus *Orthokarstenia*. In the Upper Cretaceous segments, *Orthokarstenia clavata*, *O. cretacea,* and *O. dentata* are prevalent, showing correlation with Maastrichtian assemblages within other African basins (De Klasz & Rérat, 1961; De Klasz & Rérat, 1968; Volat & Hugo, 1986). The initial appearance of *Rugoglobigerina rugosa* at 2,200 mbsf, alongside *R. rugosa, Archaeoglobigerina blowi, Planohedbergella praeriehillensis,* and *Heterohelix globulosa* between 1,416 and 2,420 mbsf, outlines a Maastrichtian interval. Above 2,420 mbsf, the presence of *P. praeriehillensis* establishes the upper limit of the Santonian, with *Muricohedbergella holmdelensis* and *Dicarinella asymetrica* also delineating this boundary (Ly, 1985). The lower Turonian depth at 2,578 mbsf is defined by the first occurrence datum (FOD) of *Praeglobotruncana cf. helvetica* and *Dicarinella algeriana*. An assemblage consisting of *Heterohelix reussi, A. blowi, A. cretacea, Marginotruncana sinuosa, M. holmdelensis, Whiteinella baltica* and *W. aprica* indicates the Santonian-Campanian boundary extending to the Upper Turonian. Frequent occurrences of *Favusella washitensis* below 2,950 mbsf characterize Lower Cenomanian and Upper Albian layers (Ly, 1985).

## Material and methods

### Sample collection

**Figure 1:**
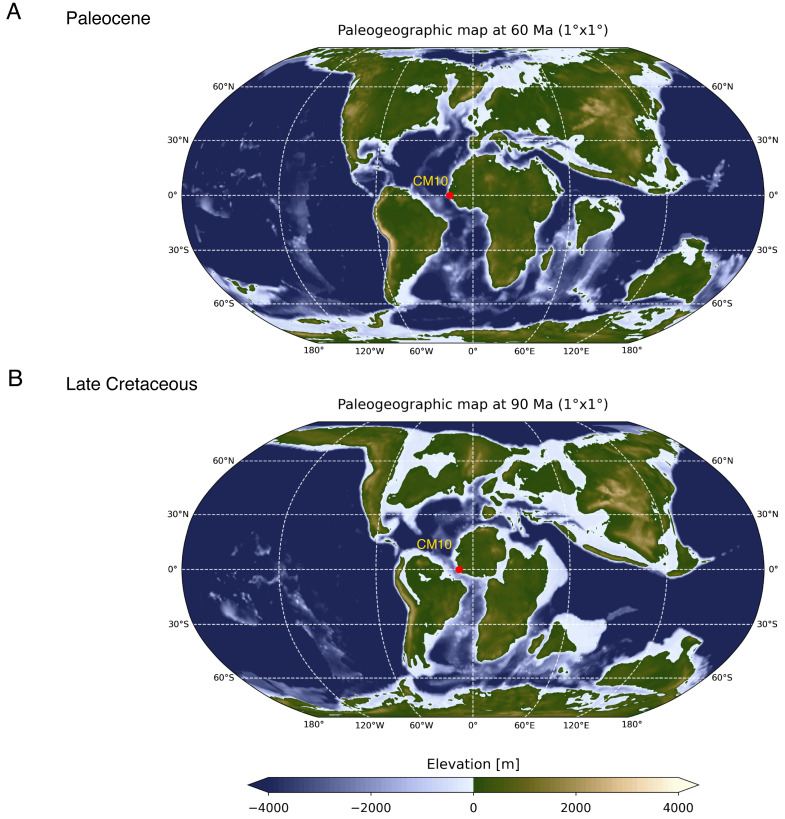
Location of offshore drillhole CM10 in Casamance area. (A) During Paleocene (60 Ma) and (B) during late Cretaceous (90 Ma). The paleogeographic maps are from Scotese & Wright (2018) placed in the paleomagnetic reference frame of Vaes et al. (2023).

Analyses were conducted on 54 dry-sieved (over 63 µm) cuttings samples retrieved from the Casamance Maritime 10 (CM10) borehole, which was drilled by a petroleum company (Senegalese Petroleum Society). The borehole reaches a depth of 3,600 mbsf and is situated in the southern Casamance offshore basin at coordinates 17°27′10″W and 12°37′45″N (Fig. 1; Portions of this text were previously published as part of a preprint in Thiam, Chaabane & De Garidel-Thoron, 2025). We prepared the sample collection stored in the geological department of Cheikh Anta Diop University in Dakar, Senegal. Samples were completely disaggregated using a buffered hydrogen peroxide solution and washed over a 63 µm sieve mesh size. The dry coarse fraction residue contains fossil planktonic, benthic and agglutinated foraminifera associated with fragments (broken shells, silicate and non-silicate detrital particles).

### Image acquisition

**Figure 2:**
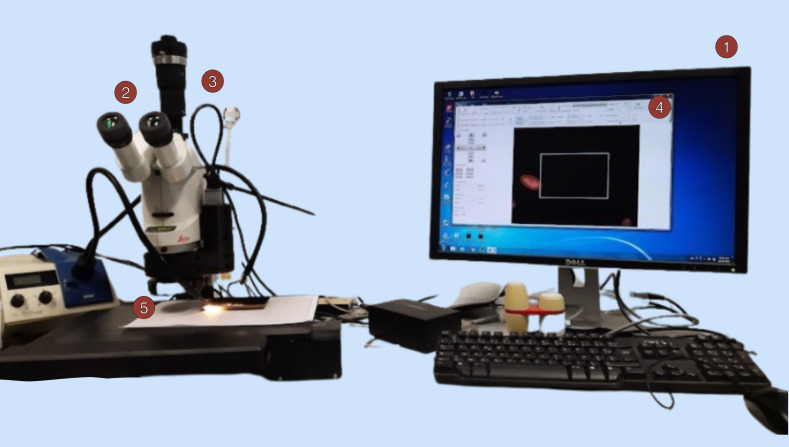
Imaging system SASHIMI developed at CEREGE. (1) Monitor, (2) Automated numerical microscope, (3) Camera, (4) Imaging software, and (5) Motorised stage.

Micropaleontological slides containing microparticles (foraminifera and fragmented species and detrital particles) were systematically imaged on a micropaleontological tray, with random orientation using an automated numerical microscope Leica MZ16A geared with a Basler camera and coupled to a motorized Leica IsoPro XY stage at CEREGE (Fig. 2). This imaging system is called “SASHIMI” and was used to image the different collected foraminifera, fragments and detrital particles from the coarse fraction. The imaging system is designed to acquire Z-stack images for particles ranging from 10 micrometers to two millimeters, with a micrometric scale resolution (2 µm). Stacked images of each particle, acquired at a vertical 70 µm step, were assembled using the Helicon Focus software (version 7.6, Helicon Soft Ltd., Kharkiv, Ukraine). Once photographed, automated segmentation was performed to identify single particles using a fixed threshold value in the grey level values of the acquired images. A total of 52,280 high resolution images of sedimentary particles (including foraminifera) were rescaled to a size of 224 × 224 pixels resolution. All images were acquired at a fixed magnification with a calibrated pixel size of 2 µm per pixel, which we used to measure test size directly from the images.

### Training dataset labelling

**Figure 3:**
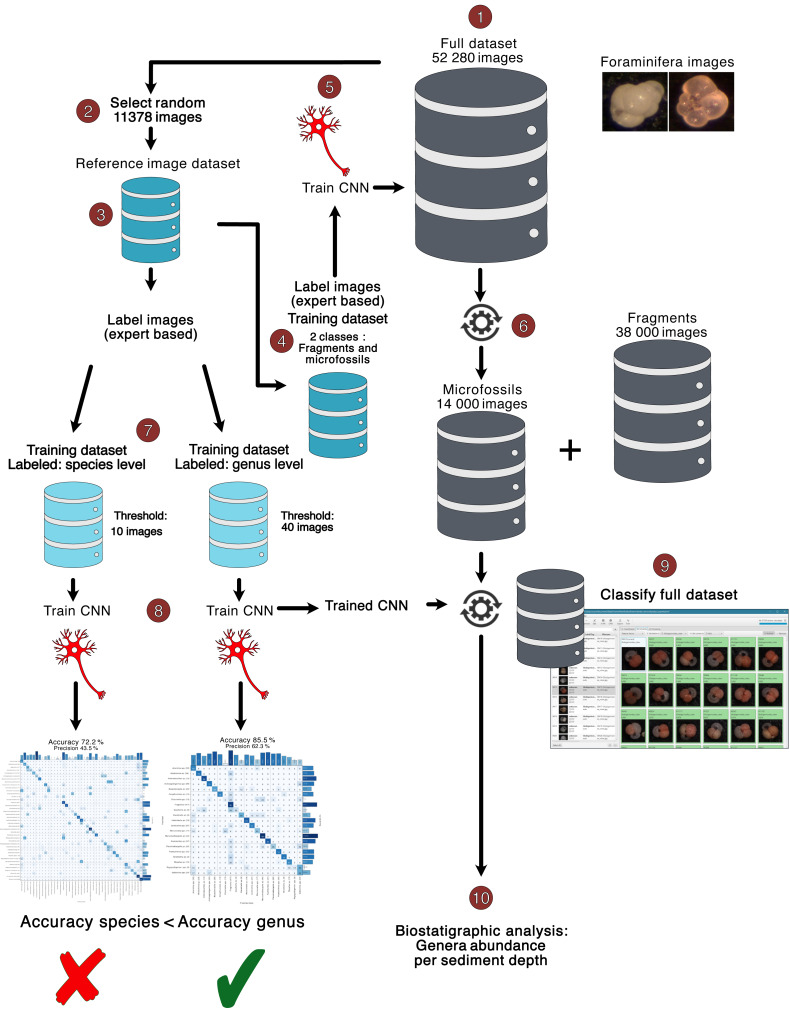
Semi-automated workflow for the acquisition and classification of foraminifera images. (1) Acquisition of images ranging all species of planktonic and benthic foraminifera and fragments; (2) Compilation of a database containing 52,280 images; (3) Random selection of images for high-level labelling into two classes (fragments and microfossils); (4) Coarse labelling of the selected images; (5) Training of a Convolutional Neural Network (CNN) using the coarse-labelled images; (6) Application of the trained CNN to label and separate fragments from the full dataset; (7) Random selection of images for fine labelling (genus and species level) using Particule-Trieur software; (8) Training of a CNN with the fine-labelled images; (9) Application of the trained CNN to label the remaining microfossil images; and (10) Generation of a confusion matrix to evaluate the CNN’s efficacy in recognizing different foraminifera species and genera.

A semi-automated workflow was developed in this study for the acquisition and classification of foraminifera images (Fig. 3). A subset of 11,378 images, the reference image dataset, was randomly selected for manual human labelling using the ParticleTrieur software *v.* 3.0.4 (Steps 2–3; Fig. 3). This subset consists of 2,469 microfossil images and 8,909 fragment images (broken shells and detrital particles). Because many species are rare and numerous taxa share similar morphological traits within the same genus, the labelling was performed hierarchically: first, images were coarsely classified into two classes, microfossils (2,469 images) and fragments (8,909 images). Microfossils were defined as shells with a recognisable test outline and chamber arrangement allowing at least genus-level identification, with minor chipping or abrasion tolerated, whereas fragments comprised broken shell pieces for which the test and chamber series could not be reconstructed, as well as non-foraminiferal detrital particles. Then, finer labelling was executed at the genus level, and finally at the species-level (Step 7; Fig. 3). All images were labelled by a single taxonomic expert (M.T.), using standard references (see below) and personal expertise in Meso-Cenozoic foraminifera. Labels were subsequently re-evaluated during model training and testing steps to correct potential errors and ensure consistency across the dataset. Our taxonomic framework follows established references (Ly, 1985; Ly & Kuhnt, 1994; Thiam, 2014; Loeblich & Tappan, 1988), complemented by nomenclatural checks against specialized databases (WoRMS Editorial Board, 2025; Foraminifera.eu Project, 2025). Notably, obtaining sufficiently crisp images for rare classes (*e.g.*, *Favusella*, *Haplophragmoides*, *Nodosaria*, *Parasubbotina*, and *Whiteinella*) was challenging. Specimens presenting heavy recrystallization or lacking diagnostic features such as a clear shell outline or chamber arrangement were excluded from the labelled dataset and assigned to a ‘non-class’ or “unclassified” category to avoid introducing noise during training. After the initial taxonomic labelling, all images were re-inspected by the same expert (M.T.) to check for morphological consistency within each taxon. Images that were obviously mislabelled, heavily recrystallized, or lacking clear diagnostic features (*e.g.*, chamber arrangement, test outline) were corrected or moved to an “unclassified” category and excluded from training.

The CNNs were trained using a categorical cross-entropy loss function between the predicted and expert labels, the standard objective function for multi-class image classification (*e.g.*, Marchant et al., 2020). For each of the three classification tasks (microfossils *vs.* fragments, species-level, genus-level), we randomly split the labelled image set per class into 80% for training and 20% for validation. Images were fed in mini-batches of 64. No independent test set was defined; all performance metrics and confusion matrices reported in this study are based on the validation subset.

### Convolutional neural network training

Once labelled, these subset images were used to train CNNs based on the ResNet-50 architecture in a transfer-learning configuration (He et al., 2016; Marchant et al., 2020). ResNet-50 is a deep residual CNN widely used for image classification; here it serves as a generic feature extractor, while transfer learning allows us to adapt its convolutional layers, originally trained on large natural-image datasets, to the specific problem of foraminifera recognition. This combination of a deep residual architecture and transfer learning has previously been shown to perform well on microfossil images (Marchant et al., 2020; Adebayo et al., 2023; Carlsson et al., 2023) and efficiently captures the complex morphological patterns and post-mortem damage present in our dataset. Following Marchant et al. (2020), the ResNet50 was used in a transfer-learning configuration, initializing the convolutional backbone with weights pre-trained on the ImageNet database (Deng et al., 2009), replacing the original 1,000-class classification layer with a randomly initialized dense layer matching the number of classes for each task, and then fine-tuning the network on our labelled foraminiferal images. All convolutional layers were kept trainable (*i.e.,* no layers were frozen), so that the entire network could adapt to the specific morphology and preservation state of our fossil foraminifera. Training was performed using the MiSo-2 v3.0.6 library written in the Python programming language (Abadi et al., 2016; Marchant et al., 2020; https://github.com/microfossil/particle-classification) and using the TensorFlow v2.15.1 library (Steps 5 and 8; Fig. 3). This study represents the first application of this MiSo/ResNet50 transfer-learning pipeline to pre-Quaternary time foraminifera time series.

Three different CNNs were trained using different labelling strategies to optimize classification performance. First, a distinction was made between fragments and microfossils, as the dataset holds many broken shells and detrital particles, which could introduce bias in CNN-based taxonomic identification. Second, a CNN was trained at the genus level to provide a broader taxonomic framework, facilitating the classification of microfossils into distinct genera and reducing misclassification among morphologically similar taxa. Finally, a third CNN was trained on the labelled dataset at the species level, enabling more precise taxonomic identification and higher-resolution differentiation of planktonic, benthic, and agglutinated foraminifera (Step 8). To further refine the training, we applied minimum image-count thresholds for each task: 10 images per class for the microfossils–fragments binary classifier, 40 images per genus, and 10 images per species. These values were chosen as a compromise between retaining rare taxa and excluding very under-represented classes that, in preliminary training runs, produced unstable loss curves and near-zero recall (Step 8). After applying these thresholds, the binary CNN (microfossils *vs.* fragments) included two classes, the genus-level CNN comprised 20 genera (nine planktonic, seven benthic and four agglutinated; see also Table 1), and the species-level CNN comprised 44 species (22 planktonic, 16 benthic and six agglutinated).

**Table 1:** Taxonomic composition of planktonic, benthic, and agglutinated foraminifera genera with their recall and species in the CM10 analysed full images dataset, along with the number of individuals identified.

**Group**	**Genera**	**Precision (%)**	**Recall (%)**	**Species**	**Number of shells**	
					**Training dataset**	**Full dataset**
Planktonic						
	Muricohedbergella sp.^*^	73.2	98	*M. holmdelensis*	212	694
	Acarinina spp.^*^	64.4	81	*A. soldadoensis*	178	607
				*A. pentacamerata*		
				*A. topilensis*		
	Planohedbergella sp.^*^	64.3	36	*P. prairiehillensis*	124	214
	Subbotina spp.^*^	36	38	*S. eocaena*	118	478
				*S. linaperta*		
				*S. triangularis*		
				*S. triloculinoides*		
	Archaeoglobigerina spp.^*^	72.2	65	*A. blowi*	100	283
				*A. cretacea*		
	Heterohelix sp.^*^	65.2	83	*H. globulosa*	91	250
	Morozovella spp.^*^	58.8	59	*M. subbotinae*	87	264
				*M. lensiformis*		
	Dicarinella spp.^*^	33.3	15	*D. asymetrica*	65	91
				*D. primitiva*		
	Rugoglobigerina sp.^*^	100	25	*R. rugosa*	40	54
Benthic						
	Praebulimina spp.^*^	81.5	80	*P. bantou*	274	594
				*P. fang*		
	*Nummulites sp.*	97.4	100	*N. rectus*	184	204
	Afrobolivina sp.^*^	86.7	93	*A. afra*	139	373
	Lenticulina spp.^*^	76.2	76	*L. trinitatensis*	106	234
				*L. degolyeri*		
				*L. rotulata*		
	*Coryphostoma sp.*	70	70	*C. midwayensis*	50	331
	*Gavelinella sp.*	33.3	12	*G. dayi*	41	41
	*Pyramidina sp.*	75	75	*P. prolixa*	40	52
Agglutinated						
	Budashevaella sp.^*^	69	77	*B. multicamerata*	130	307
	Reophax sp.^*^	81.8	60	*R. duplex*	74	210
	Ammobaculites sp.^*^	64.3	90	*A. sp1*	50	251
	*Gaudryina sp.*	100	70	*G. pyramidata*	48	57

**Notes.**

* Genera and species used for the biostratigraphic analysis

To assess the performance of the three developed CNN ResNet50 networks on the training/validation dataset, we use the standard metrics in machine learning: overall accuracy (percentage of images in the validation set that were correctly classified by the CNN), precision (percentage of images identified into a class that actually belong to the class), recall (percentage of images in a class that were correctly identified (per-class accuracy)), and F1 score (the harmonic mean of precision and recall), providing insights into the CNNs performance, and plotted the confusion matrices for each of the CNN (Fig. 3).

To quantify the relationship between training set size and classification performance, for each species and genus, the number of training images (class-specific training count) and the corresponding recall and precision on the validation subset were computed. Then simple linear regression models (ordinary least squares) between training image count and recall, and between training image count and precision, separately at the species and genus-levels were fitted. The coefficient of determination (r^2^) and associated *p*-values were reported (Fig. S5).

Additionally, we performed t-distributed Stochastic Neighbor Embedding (t-SNE) analysis (Marchant et al., 2020; Kingma & Ba, 2014; Van der Maaten & Hinton, 2008). This technique helped visualize the high-dimensional feature space of the images in a lower-dimensional (2D) space, allowing us to assess insight into the influence of morphology and imaging variability on classification performance. The t-SNE embeddings were computed in 2D using the scikit-learn TSNE implementation with random initialization and random_state = 0, a perplexity of 30 and early exaggeration of 12; all other parameters (including learning rate and number of iterations) were kept at their default scikit-learn values. The t-SNE visualization for the genus classification is shown in Fig. S4, while the t-SNE plots for species and foraminifera *vs.* fragment classifications can be found in the Zenodo repository (10.5281/zenodo.18310748).

For the three CNN trainings, the Adam optimizer was used with an initial learning rate of 0.001, and training ran for 117 epochs with early stopping for the genus, 102 epochs for the species, and 121 epochs for the foraminifera *vs.* fragment classification. Training took approximately 180 min (∼3 h) for the genus, 46 min for the species, and 170 min for the foraminifera *vs.* fragment classification on a NVIDIA Quadro RTX 6000 GPU. Both training and validation loss were tracked; LOESS-smoothed curves are provided in the supplementary material (Fig. S1). Early stopping was implemented by monitoring the validation loss and halting training when it failed to decrease by more than a small threshold for a fixed number of consecutive epochs, retaining the model parameters from the epoch with the minimum validation loss (Marchant et al., 2020; Prechelt, 2002). The trained CNNs are exported in the Open Neural Network Exchange (ONNX) format, which provides a framework-independent representation of the full ResNet50 computation graph and learned weights (ONNX Community, 2019). The network_info files store the class label list, input image size, image normalisation parameters, and key training metadata (number of epochs, early-stopping configuration, final metrics), thereby enabling fully reproducible inference and facilitating reuse or further fine-tuning of the models. The trained ONNX models, together with the network_info files, are available on Zenodo (10.5281/zenodo.18310748).

### Image inference set

The CNN trained for the binary classification (microfossils *vs.* fragments) was first used in inference mode on the full image dataset of 52,280 segmented particles to filter out fragmented shells (38,297) and detrital particles (6,211), so that only intact microfossils (7,772) were retained for subsequent analysis (Step 6). Both genus- and species-level CNNs were then used in inference mode to classify the images in this “microfossils full dataset” (Fig. 3, Step 9; Table 1). Here, the term “full dataset” refers to the complete set of 52,280 segmented particles, whereas the “microfossils full dataset” designates the subset of these images that were classified as microfossils after fragment (*i.e.,* fragment + non microfossils particles) filtering. In the MiSo-2 inference pipeline, the species-level and genus-level models are run sequentially but independently on each image, with species-level inference executed first for practical reasons rather than because of any hierarchical dependency between the two tasks. For all subsequent quantitative and biostratigraphic analyses, we use the genus-level predictions as the primary output, while species-level predictions are retained for complementary exploration and are made available in the Zenodo archive (10.5281/zenodo.18310748). This choice is motivated by the greater robustness of genus-level classification for this dataset, as detailed in ‘Dataset composition and class abundances’.

### Assessment of preservation state

To quantify the impact of post-mortem shell transformations on CNN performance, we visually assessed the preservation state of all CNN-classified microfossils in the microfossils full dataset. For each individual, the same taxonomic expert (M.T.) assigned a binary preservation label (“non-recrystallized” *vs.* “recrystallized”) based on the presence of diagenetic features such as frosted or sugary test surfaces, pervasive overgrowths, infillings of chambers, loss of wall ornamentation, and partial obliteration of sutures and chamber outlines. Specimens showing little or no diagenetic overprint and retaining sharp test margins and ornamentation were classified as non-recrystallized; specimens with clear evidence of recrystallization or strong overgrowth were classified as recrystallized. For each genus, we then counted the number of individuals in each category and expressed them as percentages of the total number of individuals for that genus. These genus-level proportions are summarized in Fig. S6 (See images in the Zenodo repository: 10.5281/zenodo.18310748).

### Biostratigraphy

We compare expert-derived biostratigraphic vertical distribution ranges of major genera of planktonic, benthic and agglutinated biozone indicator species with the CNN classified species downbore distribution. The data for this analysis were extracted from the microfossils full dataset, *i.e.,* all images classified as intact microfossils after the fragment–microfossil CNN filtering step, after applying the CNN trained on genus-level labelled images. Biozonation was determined based on planktonic key species zonal schemes using the Berggren et al. (1995), Caron (1985), Moullade (1966), Porthault (1974) and Sigal (1977) scale and benthic foraminifera established for the Meso-Cenozoic. In this framework, biostratigraphic intervals are constrained using standard datum levels and range zones, including first occurrence datum (FOD), last datum (LAD), and total range zone (TRZ) of diagnostic taxa (Berggren et al., 1995; Gradstein et al., 2012).

Benthic and agglutinated taxa provide supplement range zones based on their stratigraphic occurrences in West African (De Klasz & Gageonnet, 1965; De Klasz & Rérat, 1961; Volat & Hugo, 1986) and South American basins (Koutsoukos & Leary, 1990). For each genus, the CNN-based vertical range was defined as the interval between the shallowest and deepest depth at which at least one individual was detected, and its abundance profile with depth was extracted. These CNN-derived ranges and FOD/LOD depths were then compared to the expert biostratigraphic ranges for the same genera (Ly & Kuhnt, 1994), and agreement was evaluated based on the degree of overlap and any systematic shifts in FOD/LOD.

To compare expert-defined biostratigraphic ranges with CNN-derived depth distributions, we used two depth-by-genus matrices: (i) an expert presence–absence mask (0 = absent, 1 = present) and (ii) CNN counts of individuals per genus and depth. CNN counts were converted to presence–absence using a conservative threshold, with values <5 individuals treated as absence and values ≥ 5 as presence. Each depth–genus cell was then classified into four categories: (i) agreement–absence (expert 0, CNN 0), (ii) agreement–presence (expert 1, CNN 1), (iii) CNN-only presence (expert 0, CNN 1), and (iv) expert-only presence (expert 1, CNN 0). These categories were visualised as a categorical heatmap (Fig. S7A).

## Results

### Dataset composition and class abundances

In the full inference dataset (52,280 images), the genus-level CNN classification covered 9 planktonic genera (Table 1), with image counts ranging from 54 to 694 images (mean ≈ 326; median = 264). Two genera contained fewer than 100 images: *Dicarinella spp.* (91 images) and *Rugoglobigerina spp.* (54 images). Seven planktonic genera exceeded 200 images, with *Muricohedbergella sp.* (694 images) having the highest count. The benthic full dataset includes seven genera with image counts ranging from 41 to 594 images (mean ≈ 261; median = 234), with *Praebulimina spp.* (594 images) being the most abundant and *Gavelinella sp.* (41 images) the least abundant. For agglutinated foraminifera, image counts range from 37 to 307 (mean ≈ 201; median = 230.5), with *Budashevaella sp*. (307 images) and *Ammobaculites sp*. (251 images) being the most abundant genera.

The training dataset comprises 11,378 images, including 1,124 planktonic, 1,176 benthic, and 169 agglutinated foraminifera shell images, with an additional 8,909 images classified as “fragments” (*i.e.,* broken shells, detrital particles). For the planktonic foraminifera, the mean number of images per planktonic genus is approximately 113, with a median of 100 and a range from 40 to 212 images. Notably, *Muricohedbergella sp.* and *Acarinina spp.* are the most abundant, with 212 and 178 images, respectively (Table 1). In contrast, the least abundant genera include *Dicarinella spp.* and *Rugoglobigerina spp.*, with 65 and 40 images, respectively.

For the benthic group, the mean image count per genus is about 119, with a median of 106 and a range of 40 to 274 images (Table 1). *Praebulimina spp.* is dominant with 274 images, followed by *Nummulites sp.* (184 images). Other benthic genera, such as *Gavelinella sp.* (41 images), and *Pyramidina sp.* (40 images), have lower abundances.

**Figure 4:**
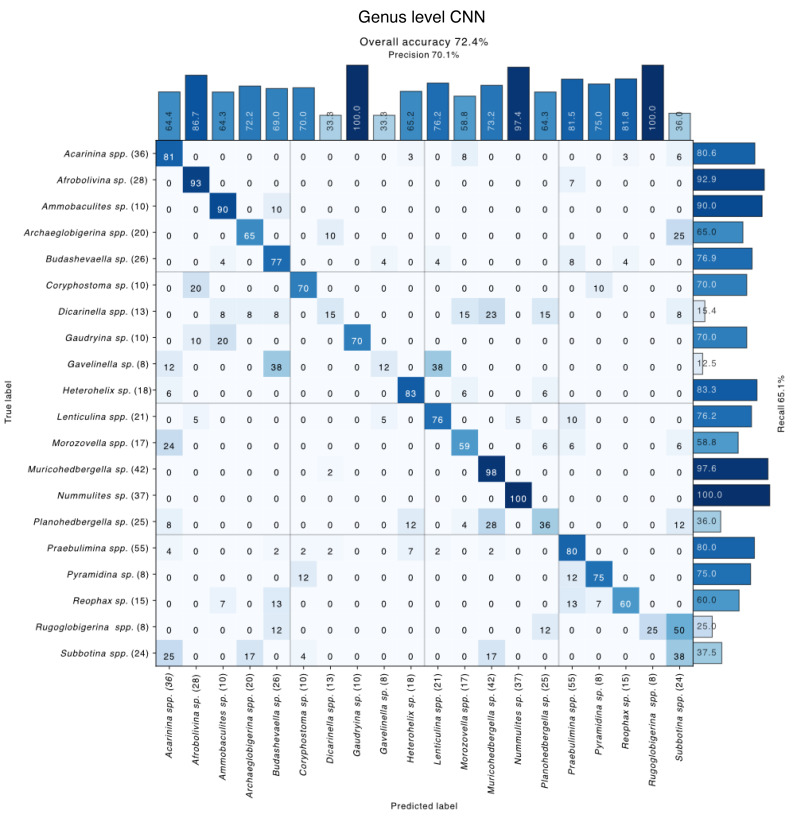
Confusion matrix of CM10 training set. The Convolutional Neural Network (CNN) classifies each image per genus in the validation set and compares it to the expert classification. The percentage of images from the class on the left (row labels) that were classified into the class on the bottom (column labels) is presented in each cell of the matrix for the validation set. The number of images in the validation set for each class is enclosed in brackets next to the respective class label; the corresponding total number of labelled images per class (training + validation) is five times this value (80% training, 20% validation).

For the agglutinated foraminifera, the mean image count per genus in this group is 68, with a median of 62 and a range from 48 to 130 images. *Budashevaella sp.* is the most abundant with 130 images, followed by *Reophax sp.*, which has 74 images. *Ammobaculites sp.* and *Gaudryina sp.* are represented by 50 and 48 images, respectively (Table 1, Fig. 4).

These class abundances in the training and full datasets provide important context for interpreting the performance metrics reported in ‘Model Evaluation’.

### Model evaluation

Three training datasets, each consisting of the same 11,378 labelled images, were used to train three distinct CNN models (microfossils *vs* fragments, genus-level, and species-level). The resulting CNNs respectively achieved overall validation accuracies of approximately 88.5% for distinguishing between microfossils and fragments, 50.2% for species-level and 72.4% for genus-level classification (Fig. 4, Figs. S2, and S3). For the binary classification of microfossils *vs.* fragments, recall reaches 86% with a macro F1-score of 87%. For the species-level model, the mean recall across classes is 40.7%, with a macro F1-score of 34% (50% when weighted by class support), reflecting the strong influence of under-represented taxa. At the genus-level, recall improves to 62.2% and the macro F1-score to 65%. A significant portion of the dataset, estimated at approximately 50%, shows visible signs of diagenetic alteration, such as recrystallization, and the under-representation of certain taxa in the training dataset (*e.g.*, species or genera with fewer than ∼20 training images; Table 1, Fig. S5A) further to likely contribute to the lower classification performance observed at the species level (Marchant et al., 2020). Because our biostratigraphic application primarily depends on correctly detecting the presence of taxa downcore, we focus in the text on overall accuracy and recall, while reporting F1-scores here for completeness. Given that genus-level classification achieved higher accuracy, recall and F1-score, and is less sensitive to class imbalance and diagenetic alteration in this dataset, subsequent analyses are conducted using the genus-level CNN results, which are better suited to our biostratigraphic interpretations than the species-level outputs.

### Taxonomic classification

**Figure 5:**
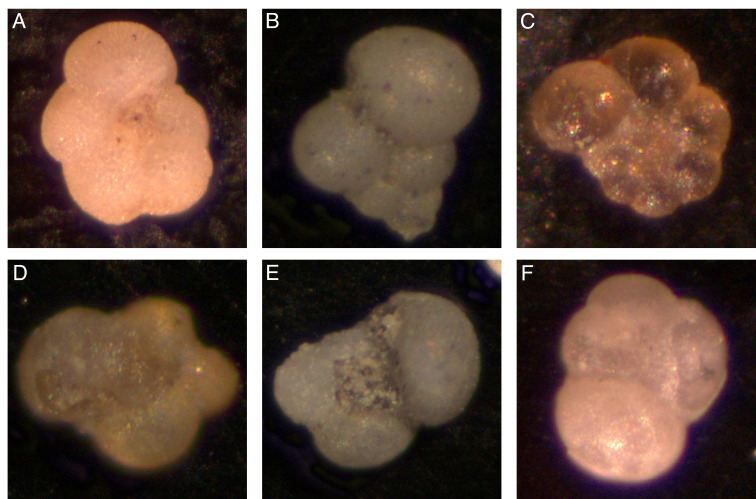
Examples of planktonic foraminifera genera. Truncorotaloidids, Heterohelicids, Hedbergellids, Rugoglobigerinids and Glogerinids images generated by the automated acquisition, processing and recognition workflow. (A) *Acarinina spp.* (1,200 m, early Maastrichtian-mid Paleocene); (B) *Heterohelix sp*. (2,200 m, late Cenomanian-early Campanian); (C) *Muricohedbergella sp.* (3,100 m, late Campanian-Turonian); (D) *Planohedbergella sp*. (3,200 m, late Cenomanian-early Campanian); (E) *Rugoglobigerina sp.* (2,200 m, late Campanian-early Maastrichtian); (F) *Subbotina spp*. (1,400 m, Late Maastrichtian-Paleocene).

**Figure 6:**
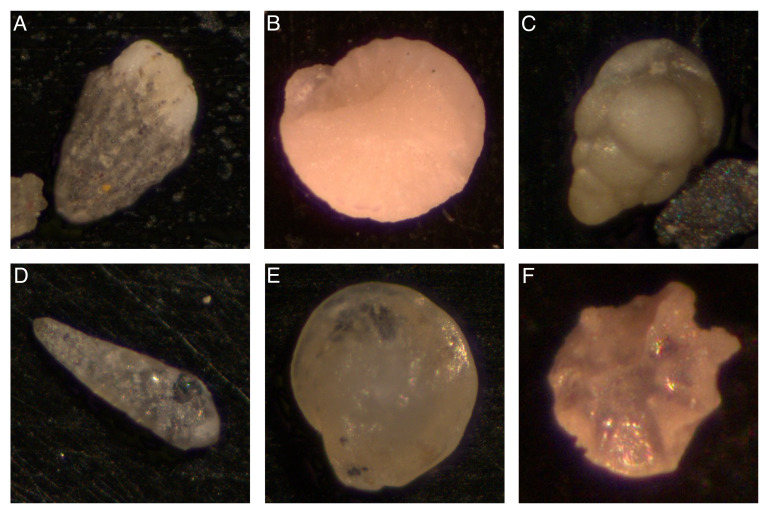
Examples of benthic foraminifera genera. Bolivinitids, Nummulites, Buliminids, Planorbulinoids and Vaginulinids images generated by the automated acquisition, processing and recognition workflow: (A) *Afrobolivina sp.* (1,900 m, Turonian-Paleocene); (B) *Nummulites sp.* (345 m, Paleocene); (C) *Praebulimina spp.* (2,198 m, Santonian-early Paleocene); (D) *Coryphostoma sp.* (3,522 m, Cenomanian); (E) *Gavelinella sp.* (2,000 m, Maastrichtian); (F) *Lenticulina spp.* (800 m, Paleocene).

The confusion matrix derived from the genus-level CNN reveals distinct performance patterns across foraminiferal groups. For planktonic taxa, *Muricohedbergella*, *Heterohelix*, *Acarinina*, and *Archaeglobigerina* achieved the highest recall values ranging from 65 to 97.6% and precision varying from 65 and 98% (Fig. 4), whereas *Planohedbergella*, *Morozovella*, *Dicarinella*, *Rugoglobigerina*, and *Subbotina* exhibited lower recall values varying from 15.4 to 58.8% and precision between 15 and 59%. In benthic foraminifera, six genera such as *Nummulites*, *Lenticulina*, *Afrobolivina*, *Praebulimina*, *Pyramidina*, and *Coryphostoma*, recorded recall values above 70%, while only *Gavelinella* performed poorly (recall: 12.5%, precision: 12%). For agglutinated foraminifera, *Ammobaculites*, *Budashevaella*, *Gaudryina*, and *Reophax* were classified with recall values exceeding 60% and precision ranging from 60% to 90% (Figs. 4, 5 and 6).

Furthermore, taxonomically similar morphotypes often lead to mislabelling, especially among genera along the same lineage. Misclassification rates are estimated at 2–38%, with certain genera more prone to confusion such as *Gavelinella sp.* often misidentified as *Budashevaella sp* (38%), *Lenticulina spp.* (38%) or *Acarinina spp.* (12%)*. Planohedbergella sp.* is mislabelled as *Muricohedbergella sp.* (28%), *Heterohelix sp*. (12%) or *Subbotina spp.* (12%). Genus *Morozovella spp.* was mostly misclassified as *Acarinina spp.* (24%), *Planohedbergella sp.* (6%) *Praebulimina spp.* (6%) or *Subbotina spp.* (6%). Otherwise, *Heterohelix sp.* is frequently misclassified as *Planohedbergella sp*. (6%), *Acarinina spp*. (6%) or *Morozovella spp*. (6%).

### Unsupervised t-SNE clustering

The t-SNE analysis on the training dataset (Fig. S4) reveals distinct clustering patterns that vary across groups. For example, within the planktonic group, *Muricohedbergella sp.* and *Planohedbergella sp.* exhibit overlapping patterns, indicating some similarity in feature space despite intrinsic differences in their respective morphology. In contrast, *Dicarinella spp.* displays a scattered distribution, suggesting significant intra-class variability. Additionally, *Archaeglobigerina sp.* tends to cluster together with *Subbotina sp.*, while *Rugoglobigerina spp.* shows a scattered pattern and occasionally overlaps with *Subbotina sp.* Interestingly, *Heterohelix sp.* forms a distinct cluster that is situated close to that of *Planohedbergella sp.*, hinting at subtle inter-group relationships. Within the planktonic taxa, *Acarinina sp.* shows bimodal clustering, part of its images group separately, whereas another portion clusters alongside *Morozovella sp*. Separately, taxa such as *Ammobaculites sp., Nummulites sp., Coryphostoma sp., Afrobolivina sp., Gaudryina sp.,* and *Pyramidina sp.* form tight, distinct clusters, indicative of their well-preserved, distinctive morphological features. In the benthic group, *Lenticulina spp.* clusters closely with *Budashevaella sp.* and *Gavelinella sp*.

### Relationship between training set size *vs.* classification performance

Although our main interpretations rely on genus-level predictions, we include species-level results here because they provide additional insight into how data scarcity and class imbalance affect model performance. At the species-level (Fig. S5A), a strong dependency was observed (*r*^2^ = 0.567, *p*-value < 0.001): species represented by fewer than 20 images (*e.g.*, *Archaeglobigerina cretacea* with 19 images and *Buliminella quadrilobata* with 16 images) exhibited near-zero recall, whereas species with more than 20 images generally could reach a recall above 70% (*e.g.*, *Muricohedbergella holmdelensis* with 235 images reaches a recall of 89.4%). Notably, exceptions exist, such as *Gaudryina pyramidata*, which achieved 85.7% recall with only 34 images.

In contrast, the genus-level analysis revealed a less pronounced relationship between count and recall (*r*^2^ = 0.234, *p*-value = 0.031; Fig. S5B). For instance, while the genus *Gavelinella sp.* is represented by only 41 images and shows a low recall of 13%, other genera such as *Muricohedbergella sp.* (212 images, 98% recall) and *Nummulites sp.* (184 images, 100% recall) consistently achieve high recall irrespective of some variability in image count. The number of images showed a moderate correlation with precision at the species level (*r^2^* = 0.43, *p* < 0.001; Fig. S5C), with higher precision for well-represented species. At the genus-level, the correlation was weaker (*r^2^* = 0.124, *p* = 0.108; Fig. S5D), with some genera achieving high precision despite limited data.

### Biostratigraphy

**Figure 7:**
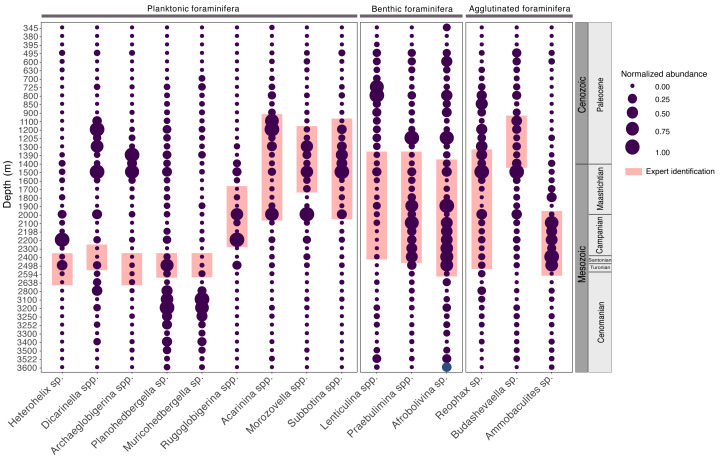
Biostratigraphic ranges of major foraminifera species identified in Meso-Cenozoic sediment samples (CM10 core). The depth distribution of foraminifera species identified by a Convolutional Neural Network (CNN) compared to the expert-based biostratigraphic classifications. Dark purple circles represent CNN identifications, where the size of the circles are proportional to the normalized abundance of each taxon at each depth. Pink rectangles indicate the stratigraphic range of key species identified by human experts based on classical biostratigraphic frameworks (Ly & Kuhnt, 1994). The CNN results are displayed across the full dataset, while expert ranges serve as a visual benchmark for comparison. Depth is plotted on the *y*-axis, with taxa along the *x*-axis.

The genus-level CNN analysis identified a range of planktonic foraminiferal genera with distinct depth and temporal distributions across the sediment (Fig. 7). *Heterohelix* sp. (2,200–2,500 m, late Cenomanian to early–mid Campanian) and *Rugoglobigerina* spp. (2,000–2,200 m, late Campanian to early Maastrichtian) show more restricted depth ranges. Some genera present broader distributions, such as *Dicarinella spp.* (1,200–2,800 m, late Cenomanian to early Paleocene). Taxa such as *Planohedbergella sp.* (2,400–3,200 m, late Cenomanian to Santonian-early Campanian) and *Muricohedbergella sp.* (3,100–3,250 m, mid-late Cenomanian) were found in older sediments, contrasting with the genera found in more recent sediments like *Archaeoglobigerina spp.* (1,300–1,500 m, early Paleocene), and *Dicarinella spp.* (1,200–1,500, early Paleocene). Genera such as *Acarinina spp.*, *Morozovella spp.*, and *Subbotina spp.* exhibited consistent overlapping distributions between 1,100–2,000 m, predominantly from the early Maastrichtian to the mid-Paleocene. For the benthic foraminifera, these species exhibited diverse depth ranges (Fig. 7). For instance, *Lenticulina spp.* were observed at relatively shallow depths of 700–900 m during the mid-Paleocene. Genera with broader and overlapping ranges included *Praebulimina spp.*, which occurred between 1,200–2,500 m from late Santonian to early Paleocene. *Afrobolivina sp.* encompasses a wide distribution (600–2,500 m, Turonian to mid Paleocene). The agglutinated foraminifera demonstrated also a combination of narrow and broad distributions (Fig. 7). *Reophax sp.* (800–2,100 m, late Campanian to the mid-Paleocene) exhibited a bimodal occurrence overlapping with the depth range of *Budashevaella sp.* that was found at shallower depths (400–2,100 m, late Campanian to the late Paleocene). Similarly, *Ammobaculites sp.* occupied depths of 2,100–2,500 m, primarily during the Turonian to late Campanian. In the top part of the core (top ≈1,100–2,000 m), where tests are generally better preserved, CNN-based depth ranges closely match the expert biostratigraphic intervals, whereas discrepancies are more frequent in the deeper, older interval where test recrystallisation and other diagenetic features are more common.

## Discussion

### Performance of the CNN classification

The binary classification between microfossils and fragments (broken shells and detrital particles) was particularly successful, with high precision and recall values (approximately 86–88%; Fig. S2), which aligns with previous studies (*e.g.*, Marchant et al., 2020; Zhong et al., 2017) emphasizing the importance of including all sedimentary particles in the training set, and to screen out the objects not of interest by a first step. Species-level classification yielded lower performance on the validation set (overall accuracy of 50.2% and an average recall of 32.8%; Fig. S3) than genus-level classification, which on the validation set achieved higher overall validation accuracy and average recall (72.4% and 65.1%, respectively; Fig. 4). This observation suggests that a coarser taxonomic approach (at the genus-level) especially where there is a significant morphological variability and post-mortem degradation helps to reduce misclassifications arising from subtle differences between closely related species or morphotypes. Consistently, the divergence between training and validation loss curves observed in some cases (Fig. S1) suggests slight overfitting, reflecting the difficulty of generalizing to new, unseen images under strong morphological variability and diagenetic alteration, and highlighting the benefit of increasing the amount of labelled data in future work.

The t-SNE plot revealed distinct clustering for genera like *Ammobaculites sp.* and *Nummulites sp.*, since their present distinct morphological features and well-preserved shells (Fig. S4). Similarly, genera such as *Afrobolivina sp.* and *Coryphostoma sp.* exhibited clear clustering. However, genera with more subtle morphological differences or those subjected to post-mortem degradation showed more overlapping and dispersed clusters. For instance, *Muricohedbergella sp.* and *Planohedbergella sp.* overlapped significantly, while *Archaeglobigerina sp.* and *Subbotina sp.* clustered closely together. *Rugoglobigerina spp.* displayed a scattered distribution, sometimes merging with *Subbotina sp.* clusters. These observations corroborate the lower performance observed in species-level identification and underscore the difficulties associated with morphologically similar or degraded specimens.

Several CNN-based studies have reported classification precisions exceeding 85–90% when diagnostic features are well preserved. In particular, models trained on specimens from top sediments to those covering the last deglacial period through the Holocene consistently achieve these high precision levels (Marchant et al., 2020; Hsiang et al., 2019; Mitra et al., 2019a; Mitra et al., 2019b).

This study shows that genus-level trained CNNs can successfully differentiate fossil foraminifera taxa, even when morphological features are compromised. The model’s robust performance, despite limited training data, supports its potential for future applications in micropaleontological research, particularly in scenarios where large annotated datasets are unavailable.

#### Planktonic foraminifera

Most of the planktonic foraminiferal genera were correctly classified by the CNN at the genus level, which we define here as genera achieving both precision and recall ≥ 60% on the validation set. Five out of nine genera meet this criterion, with precision ranging from 64.4–73.2%, recall from 65.0–97.6%, and F1-scores from 66–83% (Fig. 4; Table 1). In addition, the CNN performed best for taxa exhibiting trochospiral–globular chamber arrangements, such as *Muricohedbergella sp.,* which achieved very high recognition performance (precision 73.2%, recall 98.0%, F1-score 84%; Figs. 4 and 5).

Species like *Heterohelix sp.* and *Archaeoglobigerina sp.*, characterized by rounded trochospiral or biserial-chambers and distinct sutures (Georgescu & Abramovich, 2009; Kennett & Srinivasan, 1983), were reliably classified (83% and 65% validation recall, respectively; Figs. 4 and 5), even in the presence of minor diagenetic alterations (Fig. S6). Similarly, biserial-globular taxa, such as *Heterohelix* ***,*** achieved relatively high recognition performance, with a validation recall of 83%, benefiting from distinct morphological features like its sub-triangular test shape and strongly depressed sutures (Eicher & Worstell, 1970). However, overlapping features between biserial in apertural view and trochospiral–globular forms contributed to classification ambiguities in genera like *Heterohelix sp.*, *Muricohedbergella sp.* and *Planohedbergella sp*. (Fig. 4).

The CNN demonstrated moderate validation recall for *Acarinina spp.* (81%) and *Morozovella spp.* (59%). These results highlight that morphometric features, such as muricate ornamentation, facilitate species identification. However, substantial morphological variability, particularly in test coiling and chamber shape, limits classification accuracy (Fig. 5).

**Figure 8:**
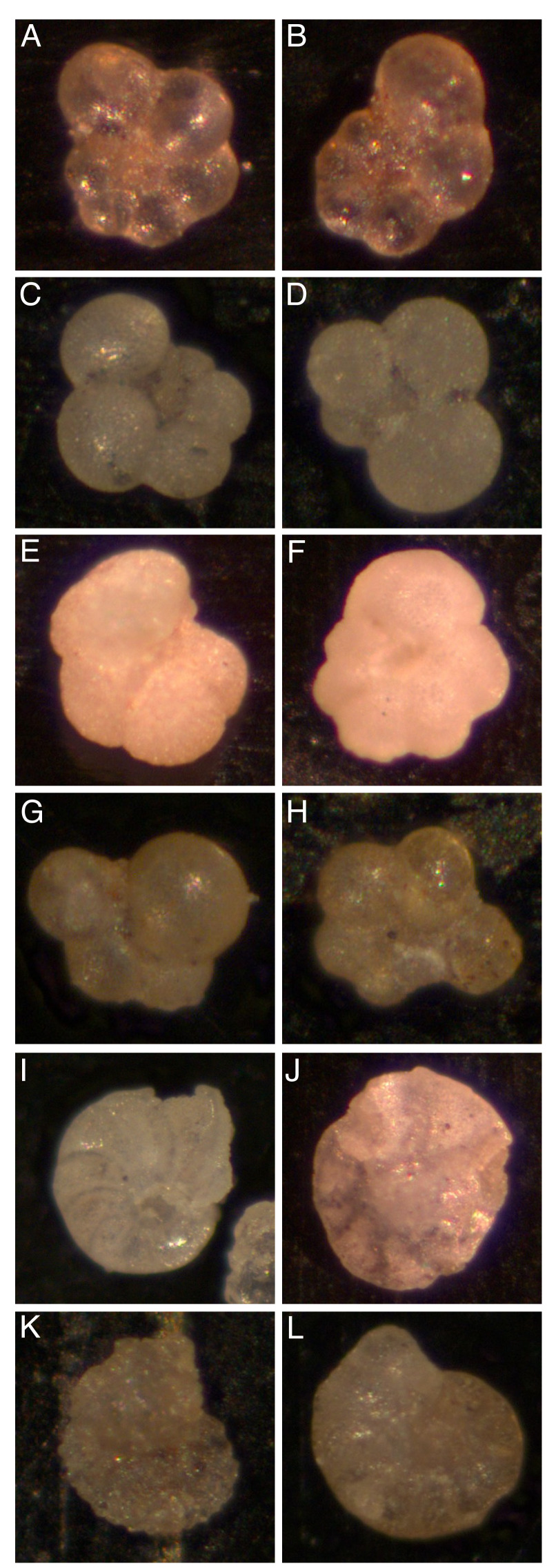
Foraminifera with similar morphologies. Examples of images depicting planktonic and benthic foraminifera identified in the CM10 samples, showcasing morphological similarities: (A) *Muricohedbergella sp.* (3,000 m, late Cenomanian-Turonian) with (B) *Planohedbergella sp.* (3,000 m, late Cenomanian-early Campanian)*;* (C) *Archaeoglobigerina spp.* (1,500 m, late Cenomanian-Turonian) with (D) *Subbotina spp.* (1,100 m, late Maastrichtian-Paleocene); (E) *Acarinina spp.* (1,100 m, late Maastrichtian-Paleocene) with (F) *Morozovella spp.* (1,200 m, late Maastrichtian-Paleocene); (G) *Heterohelix sp.* (2,200 m, late Cenomanian-Paleocene) with (H) *Planohedbergella sp.* (3,000 m, late Cenomanian-early Campanian)*;* (I) *Gavelinella sp.* (2,000 m, Maastrichtian) with (J) *Lenticulina spp.* (900 m, Paleocene); (K) *Ammobaculites sp.* (2,500 m, Turonian-Campanian) with (L) *Budashevaella sp.* (2,100 m, late Campanian-late Paleocene).

In contrast, genera with less distinctive features exhibited significantly lower recall. *Planohedbergella sp.* (36%) benefited from its compact test structure, but oblique imaging angles occasionally masked key traits (planispiral, globular chambers to radially elongate), leading to misclassification with morphologically similar taxa such as *Muricohedbergella sp., Heterohelix sp., Morozovella spp.* and *Acarinina spp.* (Figs. 4 and 8; Olsson & Hemleben, 2006). Similarly, more complex morphologies, such as *Subbotina spp.* (38%) and *Dicarinella spp.* (15%), exhibited lower classification performance due to shared traits, particularly inflated chambers and low coiling axes (Olsson et al., 2006). In addition, the genus *Subbotina spp.* presented additional challenges due to morphological similarity with *Archaeoglobigerina sp.* (Figs. 4 and 8). Both genera are characterized by globular tests with three to four chambers in the final whorl (Olsson et al., 2006).

The poorest classification performance was observed in taxa with extensive diagenetic alteration. Quartz epigenization, *i.e.,* the diagenetic replacement or overgrowth of original calcareous test material by silica (quartz), can distort chamber morphology and ornamentation, leading to systematic misidentification of *Planohedbergella* sp. aas *Muricohedbergella sp*, *Heterohelix sp.*, *Acarinina spp*. or *Subbotina spp.* (Figs. 4 and 8; Scholle & Ulmer-Scholle, 2003; Olsson & Hemleben, 2006). Beyond morphological variability, our results highlight that 2D imaging can achieve high classification accuracy for taxa with clear diagnostic features but is less effective for forms where key morphological traits are not fully captured from a single plane. For instance, the 2D imaging approach did not effectively capture the difference between petaloid chamber shapes in *Morozovella* and inflated chambers in *Acarinina*. Additionally, the plano-convex shape of the species *M. subbotinae versus* the inflated form of *M. lensiformis* led to classification errors, despite both species having similar chamber counts in the last whorl (Olsson et al., 1999). While previous studies have reported robust CNN performance with 2D imaging (Marchant et al., 2020; Hsiang et al., 2019), our findings suggest that taxon-specific factors, such as chamber inflation, coiling complexity, and diagenetic alteration, significantly influence classification outcomes. The use of 3D reconstructions, or higher resolution images with enhanced contrast (*e.g.*, Mitra et al., 2019a; Mitra et al., 2019b), in future works could further enhance recognition rates for these more challenging taxa.

#### Benthic foraminifera

Compared to planktonic foraminifera genera results, the genus-level CNN performance on benthic foraminifera classification seems better particularly for well-defined planispiral and large forms. For example, large genera such as *Nummulites sp.* consistently reached a recall of 100% (Figs. 4 and 6). Similarly, planispiral forms with involute tests, such as *Lenticulina spp*. surpassed 76% recall, likely due to their pronounced spiral turns and distinct sutural features that facilitate automated recognition.

Elongated biserial forms, including *Afrobolivina sp.* and *Coryphostoma sp.*, were recognized with intermediate to high recall (70–90%), owing to their elongate, ovate-to-lanceolate test shapes and robust costal ornamentation (Reyment, 1959; Loeblich & Tappan, 1962). Genera with elongate triserial chamber configurations, such as *Pyramidina spp.* and *Praebulimina spp.*, achieved high recall, ranging from 75% and 80%. The observed mislabelling between these taxa is attributed to their overlapping morphotypes within the Turrilinidae group (Koutsoukos & De Klasz, 2000).

Conversely, the CNN exhibited weaker classification performance for smaller, less morphologically distinctive taxa. *Gavelinella sp.,* a small planispiral form with involute convex sides, demonstrated the lowest recall (∼12%), frequently misclassified as *Budashevaella sp.*, *Lenticulina spp.*, or *Acarinina spp.* (Figs. 4 and 8). This lower performance is probably related to its compressed test morphology with consistent chamber shape, which reduces discriminative cues (Loeblich & Tappan, 2015).

Interestingly, while *Gavelinella* and *Pyramidina* are both represented by only 48 and 40 images in the training dataset, respectively, they show contrasting validation recall values (12% and 75%, respectively). This discrepancy indicates that morphological distinctiveness and lower intra-class variability (in test shape, orientation, and preservation) in *Pyramidina* can compensate for limited sample size, whereas the more homogeneous, compressed morphology of *Gavelinella*, combined with higher variability in viewing angle and preservation state, provides fewer consistent discriminative features for the CNN and results in much lower recall. In this case, the more variable and distinctive morphology of *Pyramidina*, including more pronounced chamber differentiation, likely facilitates higher recall, whereas the more homogeneous and compressed test of *Gavelinella* provides fewer discriminative features for the model.

Recent studies indicate that CNN-based classification performs well for benthic foraminifera. For example, Marchant et al. (2020) reported an overall accuracy of about 89% for benthic specimens in well preserved late Pleistocene downcore samples, attributing this to the pronounced morphological differences enabling more effective discrimination of benthic species. In contrast, Mitra et al. (2019a) and Mitra et al. (2019b) noted that variable orientations in elongated globular planktonic shells (umbilical *versus* spiral views) complicate classification. Moreover, Yayan & Yayan (2023) demonstrated that a custom CNN achieved test accuracies above 70% on benthic foraminifera.

#### Agglutinated foraminifera

Agglutinated foraminifera, exemplified by *Gaudryina*, *Ammobaculites*, *Budashevaella*, and *Reophax*, display distinctive features such as diagenetic lodges, coarsely arenaceous tests, inflated chambers, and faintly depressed sutures. Our findings indicate that CNNs reliably classify taxa with heavy test biomineralization, as shown by high recall values (77–90%) for *Budashevaella sp.* and *Ammobaculites sp.* (Fig. 8). In contrast, genera with a lower proportion of agglutinated material relative to the cement, such as *Reophax sp.* and *Gaudryina sp.* are less consistently recognized (60–70%), likely due to variations in shell ornamentation. This reduced performance for *Reophax sp*., which includes two morphotypes (coarsely *versus* finely agglutinated walls), suggests that the absence of distinct test ornamentation compromises CNN accuracy. These observations reinforce long-standing taxonomic challenges in identifying Textularids based on microstructure and test perforations (Hofker, 1976; Reyment, 1969; Loeblich & Tappan, 1988; Hemleben et al., 1990). Our study confirms the diagnostic value of arenaceous microstructures and biogenetic calcite in agglutinated foraminiferal classification, as previously proposed by Bender (1989), Brönnimann & Whittaker (1988), Loeblich & Tappan (1988) and Hohenegger (1990). Occasional misclassifications are observed between genera within the same subclass Textulariana (*e.g.*, *Budashevaella vs. Ammobaculites*) or with morphologically similar biserial “hyaline” Planorbulinoidea (*e.g.*, *Gavelinella*), echoing earlier sources of taxonomic confusion (Schwager, 1877).

### Implications for the biostratigraphy: limits and recommendations

The application of a CNN for analyzing foraminiferal specimens from the Upper Cretaceous Casamance basin reveals both the promise and limitations of automated biostratigraphic approaches. The basin itself is characterized by sparse planktonic foraminiferal diversity and signs of test etching and dissolution (Ly & Kuhnt, 1994; Thiam, 2014), conditions that challenge traditional taxonomic methods which rely on manual picking and visual identification of foraminifera under the microscope by specialist biostratigraphers. Our results showed that the CNN on genera level correctly identified five out of nine planktonic genera and effectively recognized most benthic (two of three)) and agglutinated (two of three)) taxa within their expected biozones (Fig. 7 and Fig. S7) when compared with the expert-defined distributions (Ly & Kuhnt, 1994). The categorical heatmap and summary histogram (Fig. S7) show that CNN-derived distributions broadly agree with expert biostratigraphic ranges. For most genera, total CNN–expert agreement (presence + absence) exceeds ∼70%, indicating a strong overall consistency. Disagreements are dominated by expert-only presence (CNN absence), particularly in deeper intervals affected by recrystallisation and poorer preservation, whereas CNN-only presences are comparatively rare and were mainly limited to isolated occurrences in *Planohedbergella sp.* and *Muricohedbergella sp.* (Fig. S7). This confirms that discrepancies mainly reflect under-detection by the CNN rather than systematic over-extension of stratigraphic ranges. The CNN performance was particularly robust for species from younger, better-preserved sediments. Genera such as *Acarinina sp*. and *Morozovella sp*. recorded between 1,100 and 2,000 mbsf (Fig. 7) were consistently identified within their expected biostratigraphic intervals, in agreement with expert data. In contrast, the analysis of older sediments proved more challenging. For example, while *Rugoglobigerina spp.* was accurately recognized within its biozone (2,000–2,200 m), species with subtler or overlapping morphological traits, such as *Planohedbergella sp.* and *Muricohedbergella sp.*, tended to be misclassified (Nanni et al., 2023). These issues are further compounded by factors such as image angles that may obscure key diagnostic features like suture depressions, chamber inflation and chamber number in the last whorl (Olsson & Hemleben, 2006; Nanni et al., 2023). Misclassifications also emerged from morphological similarities among closely related species. For instance, while the CNN performed well on species such as *Heterohelix sp*., it occasionally confused *Archaeoglobigerina sp*. and *Dicarinella spp*. with *Subbotina spp*. (Fig. 7) a problem that echoes the challenges noted in earlier literature (Eicher & Worstell, 1970; Koutsoukos & De Klasz, 2000). Such shifts in biozonation could also be due to contamination during sample processing or the difficulty in distinguishing subtle variations in species morphology (Ly & Kuhnt, 1994; Thiam, 2014).

In light of these results, our study shows that CNN-based methodologies can classify poorly preserved foraminifera, complementing traditional taxonomic approaches. However, the approach can be improved with further work. For instance, using multi-dimensional imaging techniques could capture a broader range of morphological details, thus improving the accuracy of automated classifications. In our study, poor sample preservation, particularly the high rate of recrystallization, made species-level morpho-identification challenging (Fig. 4; Fig. S6). Our dataset, which is strongly affected by diagenetic alterations, the CNN’s ability to resolve the fine morphological details required for accurate species-level classification was significantly impaired. Across the genera included in our biostratigraphic analysis, a substantial fraction of individuals shows clear post-mortem shell transformations. At the planktonic level, the proportion of recrystallized tests per genus ranges from ∼5% (*Archaeoglobigerina* spp.) to >80% (*Muricohedbergella* sp.), with most genera falling between ∼15% and 35% (Fig. S6). Among benthic genera, the proportion of recrystallized individuals spans from <10% (*Gavelinella* sp.) to ∼60% (*Pyramidina* sp.), with intermediate values for *Praebulimina* spp., *Afrobolivina* sp. and *Lenticulina* spp. Overall, roughly half of the analysed individuals exhibit moderate to strong recrystallization features, confirming that our dataset is dominated by variably altered tests rather than pristine material. Our current dataset does not allow us to compare outcomes from images without diagenetic alterations to those with altered shells without substantially reducing the number of usable images, a reduction that would compromise model training and validation. Moreover, the presence of underrepresented classes further challenges the model’s ability to generalize. At the same time, our analysis confirms that training-set size also influences performance. Species represented by fewer than ∼20 images often show near-zero recall, and recall increases significantly with the number of training images at the species level (*r*^2^ = 0.57, *p* < 0.001; Fig. S5). These relationships indicate a genuine effect of class imbalance on model performance.

Future work should focus on expanding the training dataset with more balanced class representation, achieved either by increasing sample collection or through advanced class-balancing techniques such as re-sampling or loss re-weighting (He & Garcia, 2009; Buda, Maki & Mazurowski, 2018; Johnson & Khoshgoftaar, 2019). This would enhance the model’s generalization and classification performance across all species. Furthermore, controlling for preservation state will allow for a more precise assessment of each factor’s influence on classification accuracy. Image quality and training-set size play a crucial role in CNN performance (LeCun, Bengio & Hinton, 2015; Hsiang et al., 2019; Marchant et al., 2020), and thus, by combining high-quality images, a balanced and expanded training dataset, and traditional paleontological expertise, future studies can achieve more precise biostratigraphic interpretations. These improvements are essential for reducing misclassifications resulting from sample contamination and challenges in distinguishing subtle morphological differences.

While previous work has often focused on recent or well-preserved samples, especially in modern paleoceanographic studies and late Quaternary assemblages (Hsiang et al., 2019; Marchant et al., 2020), our results show that CNNs can lead to satisfactory classifications even under challenging conditions. The robustness of the approach is further supported by the biostratigraphic outcomes, which are consistent with established zonation schemes (*e.g.*, Ly & Kuhnt, 1994; Thiam, 2014), thereby opening the possibility for broader applications in long-term stratigraphic studies. These findings also suggest that deep learning models may help refine existing taxonomic frameworks, particularly by highlighting inconsistencies or ambiguities in traditional classifications under suboptimal conditions.

## Conclusion

This study highlights the potential of CNNs for the automated classification over long sedimentary sequences as shown in our test case on Meso-Cenozoic foraminifera from the West African margin. The results show that CNNs are particularly effective at genus-level classification, outperforming species-level identification due to the ability to reduce misclassifications driven by morphological variability and post-mortem degradation in closely related species. While genera with distinct morphological features, such as *Muricohedbergella* and *Heterohelix*, were successfully identified, taxa with more complex or variable traits, like *Subbotina* and *Dicarinella*, presented challenges, particularly in the presence of diagenetic alterations. In benthic foraminifera, *Nummulites* was easily classified, while smaller genera like *Gavelinella* proved more difficult to differentiate. Our results also underscore the current limitations of 2D imaging, particularly for taxa with complex coiling and chamber arrangements that are not fully captured in a single plane with annular lighting.

These findings highlight the importance of increasing the number of images per species to further enhance the classification accuracy. Future work should explore the integration of 3D imaging techniques and multi-modal datasets to improve species-level differentiation and reduce misclassification between closely related taxa.

By leveraging deep learning for taxonomic identification, this study provides a valuable step toward automating the processing of micropaleontological samples, facilitating large-scale paleoenvironmental and biostratigraphic reconstructions.

Beyond foraminifera, the application of CNNs can be extended to other microfossil groups such as ostracods, and more broadly to other plankton-related image datasets (Athersuch et al., 1994; Weldrick, 2022).

##  Supplemental Information

10.7717/peerj.21437/supp-1 Supplemental Information 1 Supplemental Figures **Fig S1**: LOESS-smoothed curves of training and validation loss over the epochs for the three classifications. The curves depict the performance of the CNN model during training for the genus (117 epochs), species (102 epochs), and foraminifera vs. fragment (121 epochs) classifications. The training and validation loss are plotted over the respective epochs for each classification task. LOESS smoothing was applied to both training and validation loss for clarity. **Fig S2**: Confusion matrix for the CM10 training set for a two-class classification. The matrix shows the percentage of images from each expert-defined class (microfossils and fragments, indicated by the row labels) that were classified by the Convolutional Neural Network (CNN) into each predicted class (indicated by the column labels) for the validation set. The number of images in the validation set for each class is indicated in brackets next to the class label, and the total number of images in the training set is four times this amount. **Fig S3**: Confusion matrix for the species-level training set. The Convolutional Neural Network (CNN) classifies each image in the validation set at the species level and compares the predictions with expert-assigned labels. In each cell of the matrix, the percentage of images from the species indicated by the row that were classified into the species indicated by the column is displayed. The number of images in the validation set for each species is shown in brackets next to the species label, with the total number of images in the training set being three times this amount. **Fig S4**: Traning dataset t-SNE analysis on the effect of imaging angles on micropaleontological classification. t-SNE visualization of foraminifera and fragmented particles reveals distinct clustering for some genera based on imaging angles, suggesting that angle variation influences classification.**Fig S5**: Training set size vs. classification performance. (A–B) Recall vs. number of training images for species (A) and genera (B). Recall increases with sample size, especially at species - level (r^2^ = 0.57, p-value ¡ 0.001); genus-level performance is less affected (r^2^ = 0.234, p-value = 0.031). (C–D) Precision vs. number of training images for species (C) and genera (D). Precision moderately correlates with sample size at species level (r^2^ = 0.43, p-value ¡ 0.001), but not significantly at genus - level (r^2^ = 0.124, p-value = 0.108). Red lines show linear fits; gray areas indicate confidence intervals.**Fig S6**: Recrystallization state by genus in planktonic and benthic foraminifera. Proportion of recrystallized versus non-recrystallized individuals per genus for (A) benthic and (B) planktonic foraminifera included in the biostratigraphic analysis. Bars show the percentage of specimens in each genus classified as having clearly recrystallized tests versus those retaining non-recrystallized preservation; genera are ordered within each panel by increasing proportion of recrystallized individuals.**Fig S7**: Quantitative comparison between expert-defined and CNN-derived biostratigraphic ranges. (A) Histogram showing, for each genus, the percentage of depth levels where CNN and expert classifications agree, including both joint presence (CNN = present, expert = present) and joint absence (CNN = absent, expert = absent), relative to all possible depth–genus combinations. CNN presence was defined using a threshold of ≥ 5 individuals, with lower counts treated as absence. (B) Heatmap showing, for each depth and genus, the categorical match between expert interpretation and CNN predictions after binarisation. Expert occurrences were coded as presence when the expert mask equals 100 (absence = 0). CNN occurrences were coded as presence when the inferred count is ≥ 5 individuals (counts ¡5 treated as absence). Each cell therefore falls into one of four categories: both absent (expert = 0, CNN = 0), both present (expert = 1, CNN = 1), CNN only (expert = 0, CNN = 1), or expert only (expert = 1, CNN = 0).

## Additional Information and Declarations

### Data Availability

The following information was supplied regarding data availability: The raw data is available at Zenodo: - Thiam, M., Chaabane, S., & de Garidel-Thoron, T. (2026). Automated Recognition of Meso-Cenozoic Foraminifera from Senegalese Sedimentary Deposits Using Convolutional Neural Networks (Version 02). Zenodo. https://doi.org/10.5281/zenodo.18310748
