# Supplementary material for: Automated recognition of Meso-Cenozoic foraminifera from Senegalese sedimentary deposits using convolutional neural networks
Source: PeerJ. 2026 Jun 26;14:e21437. doi: 10.7717/peerj.21437 (PMC13312969; doi:10.7717/peerj.21437)
Supplement: Supplemental Information 1 — Fig S1: LOESS-smoothed curves of training and validation loss over the epochs for the three classifications. The curves depict the performance of the CNN model during training for the genus (117 epochs), species (102 epochs), and foraminifera vs. fragment (121 epochs) classifications. The training and validation loss are plotted over the respective epochs for each classification task. LOESS smoothing was applied to both training and validation loss for clarity. Fig S2: Confusion matrix for the CM10 training set for a two-class classification. The matrix shows the percentage of images from each expert-defined class (microfossils and fragments, indicated by the row labels) that were classified by the Convolutional Neural Network (CNN) into each predicted class (indicated by the column labels) for the validation set. The number of images in the validation set for each class is indicated in brackets next to the class label, and the total number of images in the training set is four times this amount. Fig S3: Confusion matrix for the species-level training set. The Convolutional Neural Network (CNN) classifies each image in the validation set at the species level and compares the predictions with expert-assigned labels. In each cell of the matrix, the percentage of images from the species indicated by the row that were classified into the species indicated by the column is displayed. The number of images in the validation set for each species is shown in brackets next to the species label, with the total number of images in the training set being three times this amount. Fig S4: Traning dataset t-SNE analysis on the effect of imaging angles on micropaleontological classification. t-SNE visualization of foraminifera and fragmented particles reveals distinct clustering for some genera based on imaging angles, suggesting that angle variation influences classification.Fig S5: Training set size vs. classification performance. (A–B) Recall vs. number of training images for spe [file peerj-14-21437-s001.pdf]

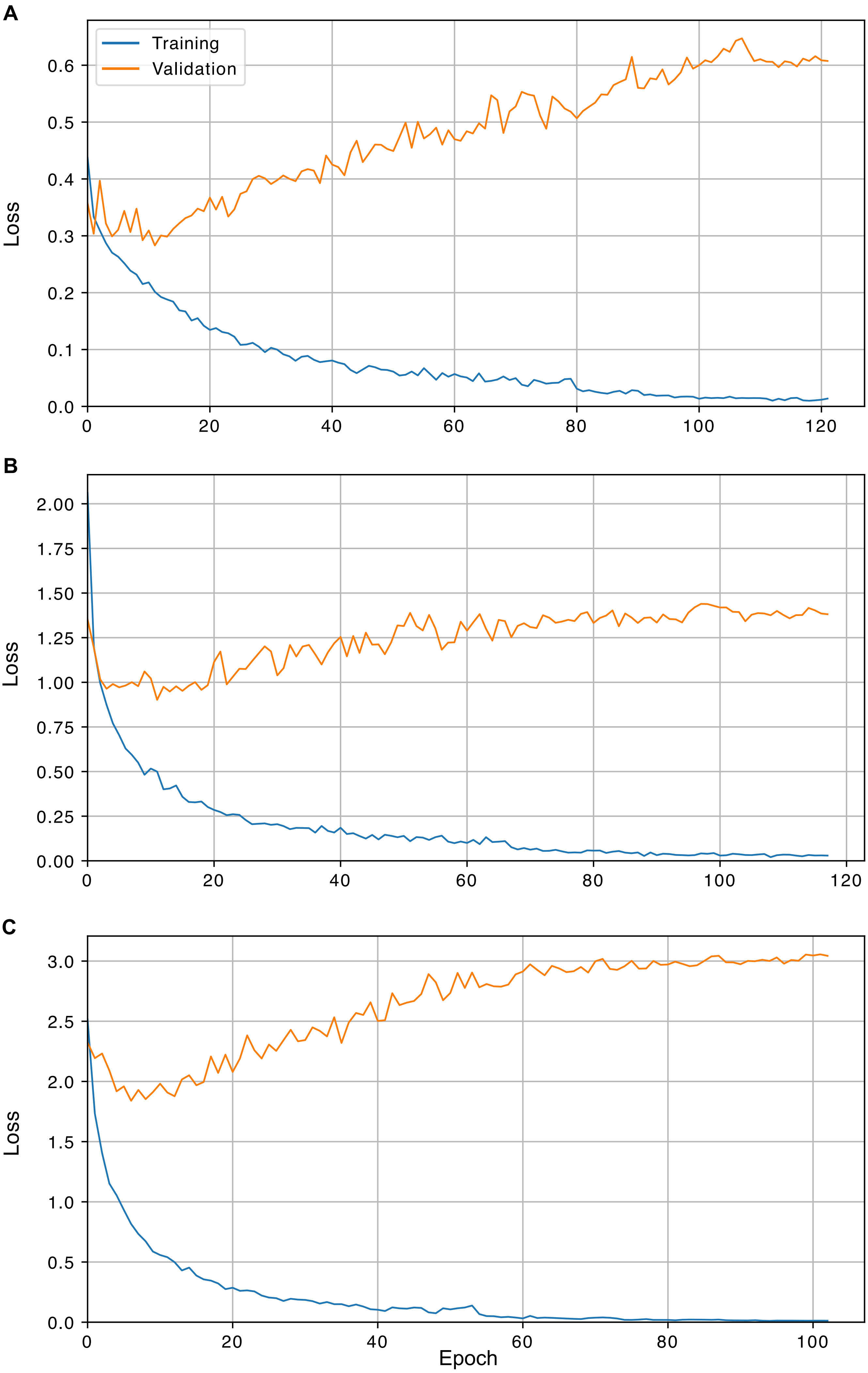
A
B
C
Training
Validation
Loss
Epoch
0.0
0.1
0.2
0.3
0.4
0.5
0.6
0.00
0.25
0.50
0.75
1.00
1.25
1.50
1.75
2.00
0.0
0.5
1.0
1.5
2.0
2.5
3.0
0
20
40
60
80
100
120

Fig. S1

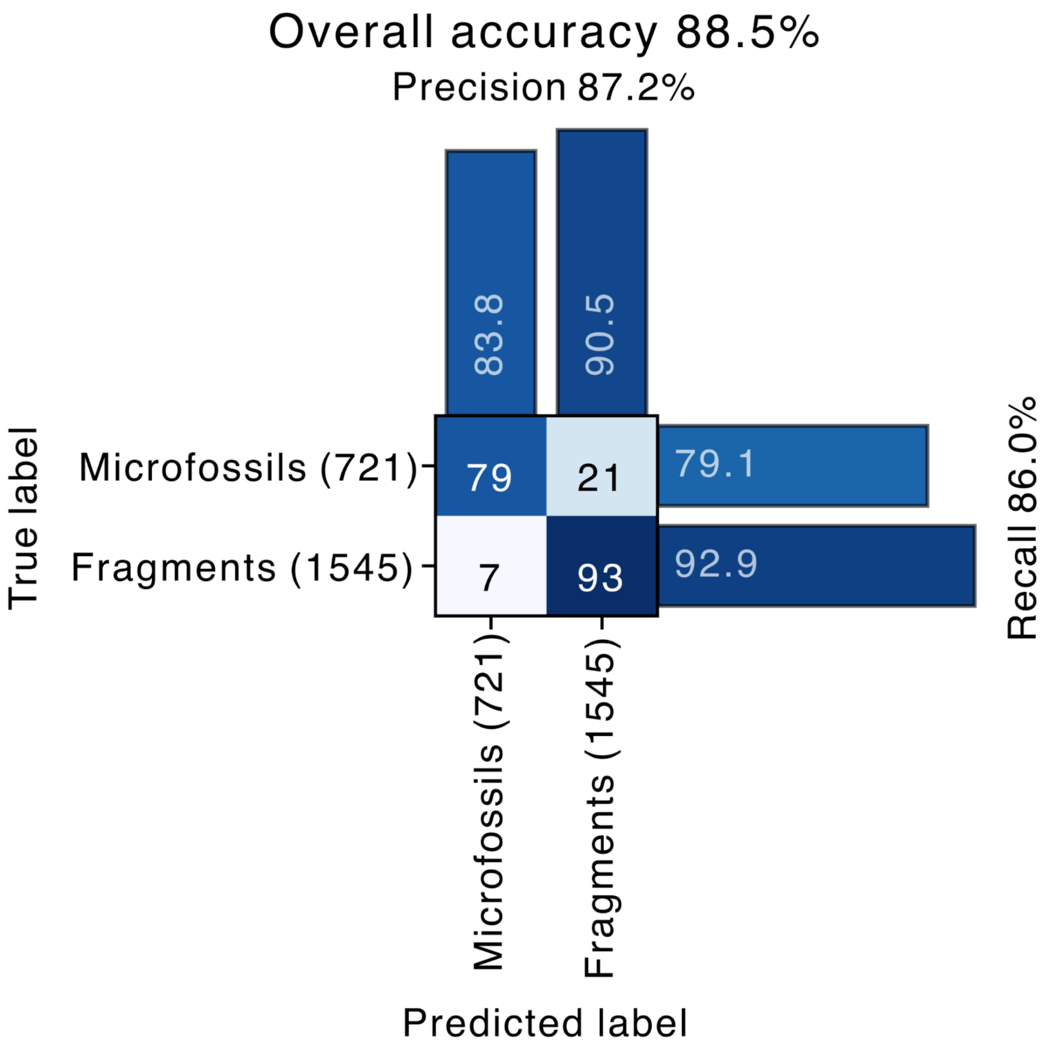

Overall accuracy 88.5%
Precision 87.2%
83.8
90.5
True label
Microfossils (721)
Fragments (1545)
79
21
7
93
79.1
92.9
Recall 86.0%
Microfossils (721)
Fragments (1545)
Predicted label

Fig. S2

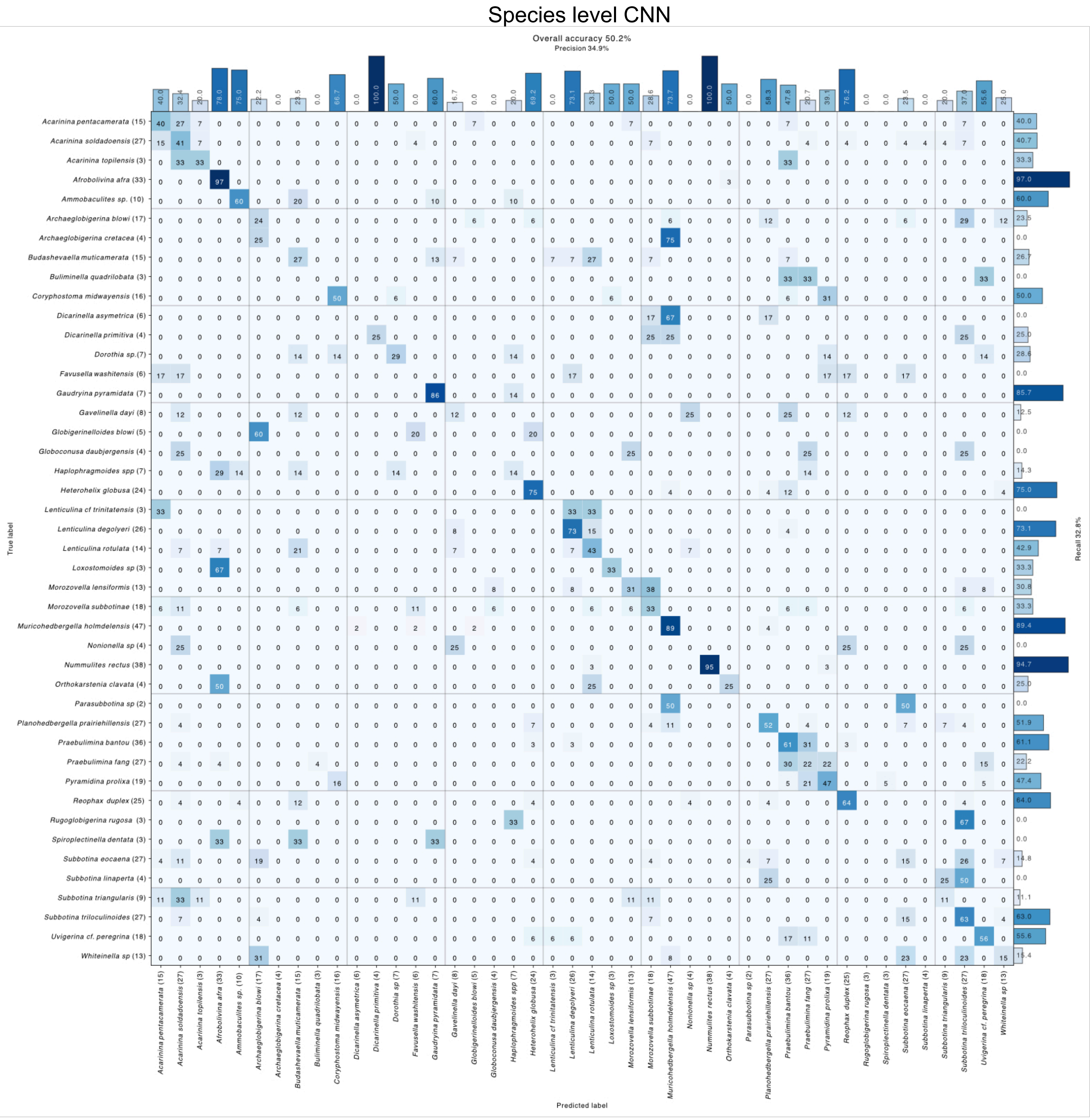
Species level CNN
Overall accuracy 50.2%
Precision 34.9%
True label
Predicted label
Recall 32.8%
Acarinina pentacamerata (15)
Acarinina soldadoensis (27)
Acarinina topilensis (3)
Afrobolivina afra (33)
Ammobaculites sp. (10)
Archaeglobigerina blowi (17)
Archaeglobigerina cretacea (4)
Budashevaella muticamerata (15)
Buliminella quadrilobata (3)
Coryphostoma midwayensis (16)
Dicarinella asymetrica (6)
Dicarinella primitiva (4)
Dorothia sp.(7)
Favusella washitensis (6)
Gaudryina pyramidata (7)
Gavelinella dayi (8)
Globigerinelloides blowi (5)
Globoconusa daubjergensis (4)
Haplophragmoides spp (7)
Heterohelix globusa (24)
Lenticulina cf trinitatensis (3)
Lenticulina degolyeri (26)
Lenticulina rotulata (14)
Loxostomoides sp (3)
Morozovella lensiformis (13)
Morozovella subbotinae (18)
Muricohedbergella holmdelensis (47)
Nonionella sp (4)
Nummulites rectus (38)
Orthokarstenia clavata (4)
Parasubbotina sp (2)
Planohedbergella prairiehillensis (27)
Praebulimina bantou (36)
Praebulimina fang (27)
Pyramidina prolixa (19)
Reophax duplex (25)
Rugoglobigerina rugosa (3)
Spiroplectinella dentata (3)
Subbotina eocaena (27)
Subbotina linaperta (4)
Subbotina triangularis (9)
Subbotina triloculinoides (27)
Uvigerina cf. peregrina (18)
Whiteinella sp (13)

Fig. S3

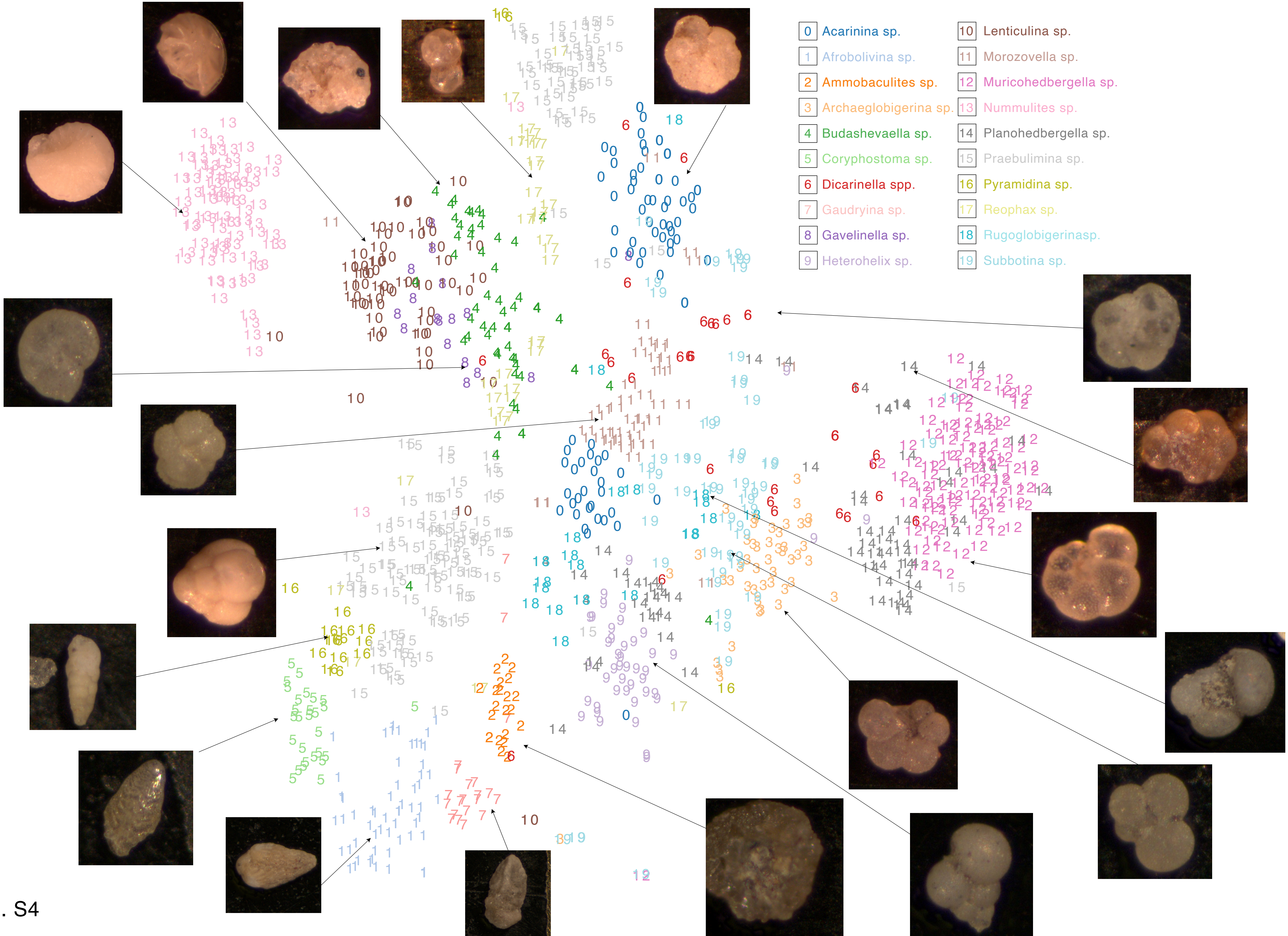
0 Acarinina sp.
1 Afrobolivina sp.
2 Ammobaculites sp.
3 Archaeglobigerina sp.
4 Budashevaella sp.
5 Coryphostoma sp.
6 Dicarinella spp.
7 Gaudryina sp.
8 Gavelinella sp.
9 Heterohelix sp.
10 Lenticulina sp.
11 Morozovella sp.
12 Muricohedbergella sp.
13 Nummulites sp.
14 Planohedbergella sp.
15 Praebulimina sp.
16 Pyramidina sp.
17 Reophax sp.
18 Rugoglobigerinasp.
19 Subbotina sp.

Fig. S4

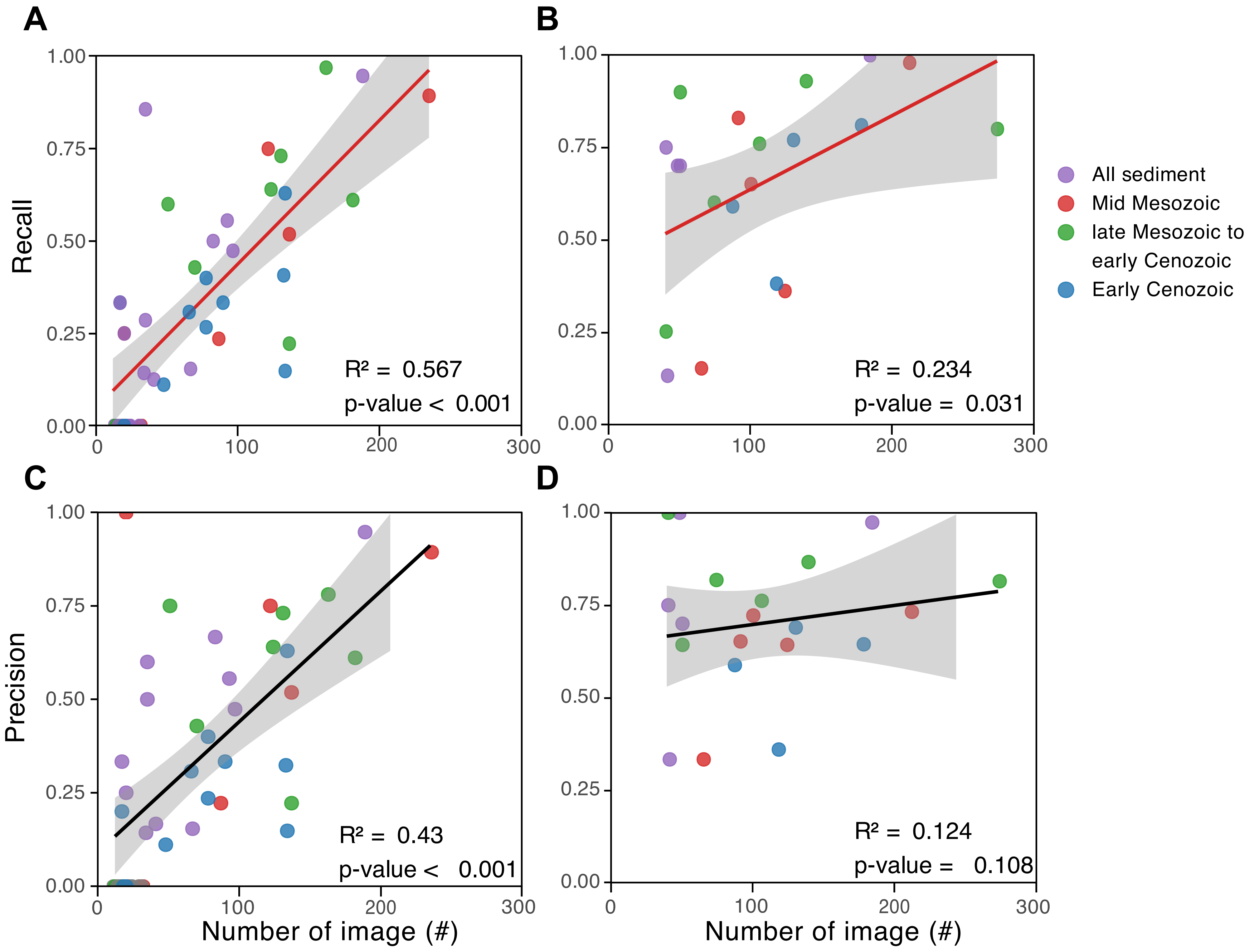
A
B
C
D
Recall
Precision
Number of image (#)
Number of image (#)
1.00
0.75
0.50
0.25
0.00
0
100
200
300
R² = 0.567
p-value < 0.001
R² = 0.234
p-value = 0.031
R² = 0.43
p-value < 0.001
R² = 0.124
p-value = 0.108
All sediment
Mid Mesozoic
late Mesozoic to early Cenozoic
Early Cenozoic

Fig. S5

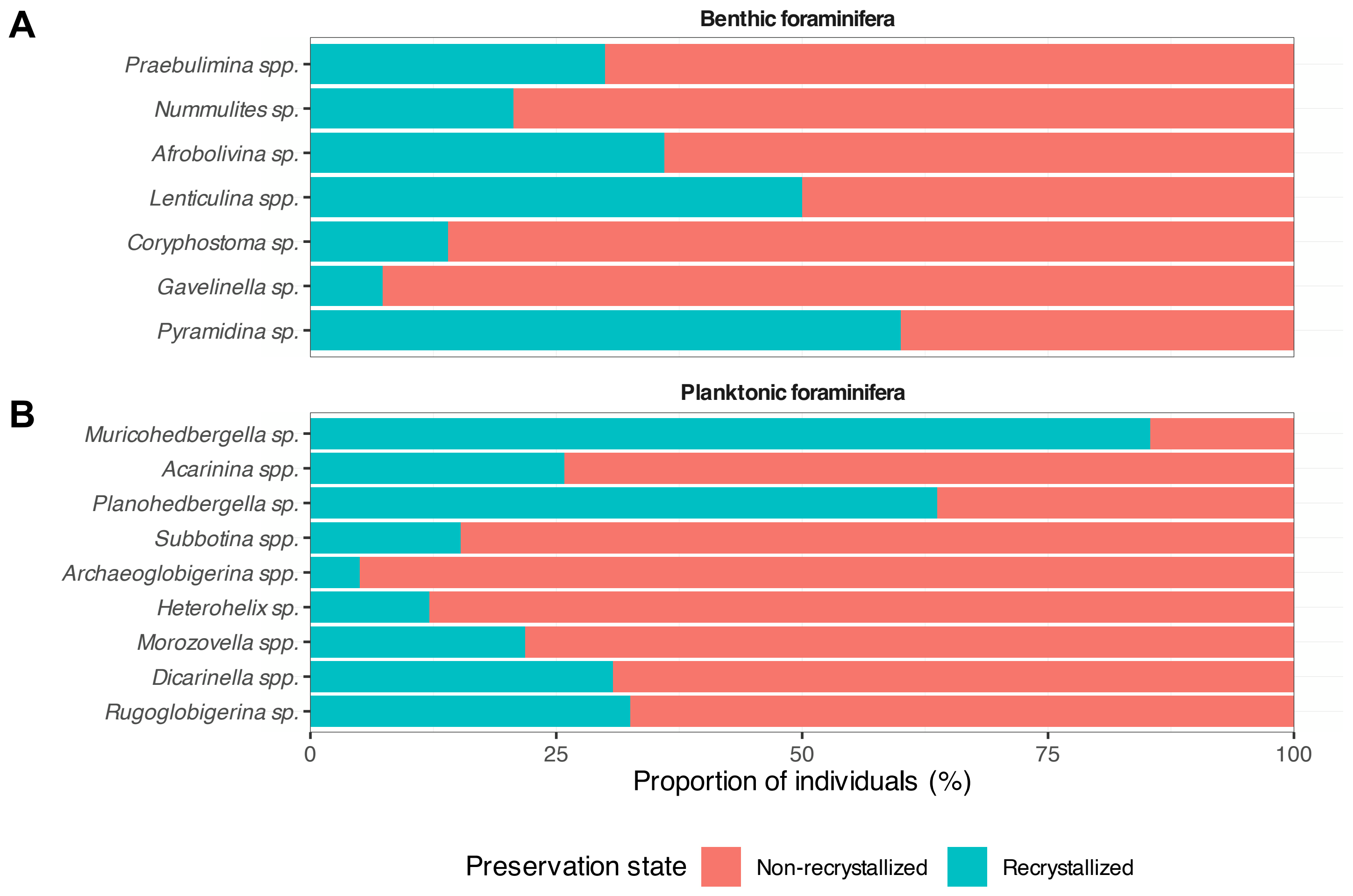
A
Benthic foraminifera
Praebulimina spp.
Nummulites sp.
Afrobolivina sp.
Lenticulina spp.
Coryphostoma sp.
Gavelinella sp.
Pyramidina sp.
B
Planktonic foraminifera
Muricohedbergella sp.
Acarinina spp.
Planohedbergella sp.
Subbotina spp.
Archaeoglobigerina spp.
Heterohelix sp.
Morozovella spp.
Dicarinella spp.
Rugoglobigerina sp.
0
25
50
75
100
Proportion of individuals (%)
Preservation state
Non-recrystallized
Recrystallized

Fig. S6

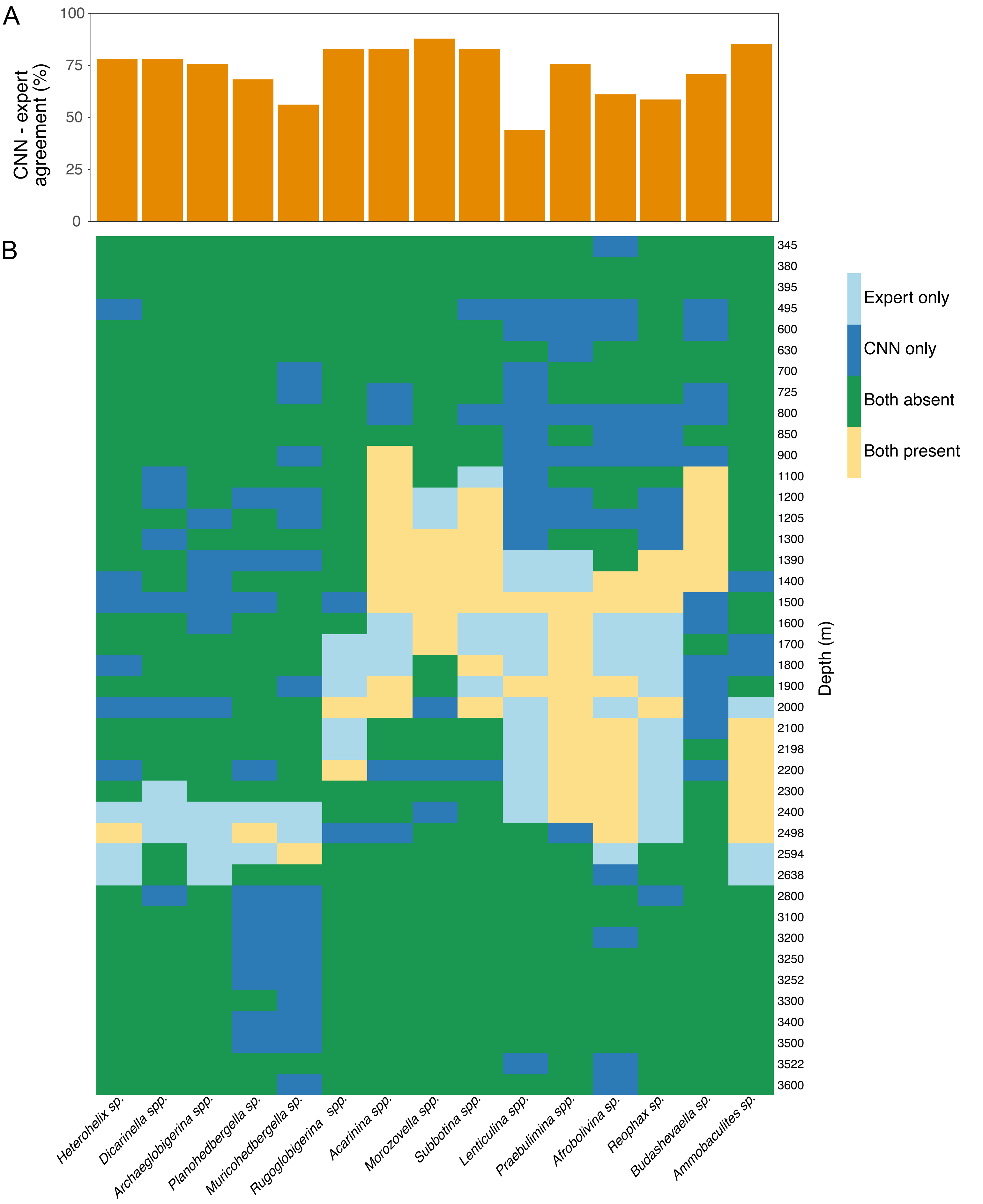
A
CNN - expert agreement (%)
100
75
50
25
0
B
345
380
395
495
600
630
700
725
800
850
900
1100
1200
1205
1300
1390
1400
1500
1600
1700
1800
1900
2000
2100
2198
2200
2300
2400
2498
2594
2638
2800
3100
3200
3250
3252
3300
3400
3500
3522
3600
Depth (m)
Expert only
CNN only
Both absent
Both present
Heterohelix sp.
Dicarinella spp.
Archaeglobigerina spp.
Planohedbergella sp.
Muricohedbergella sp.
Rugoglobigerina spp.
Acarinina spp.
Morozovella spp.
Subbotina spp.
Lenticulina spp.
Praebulimina spp.
Afrobolivina sp.
Reophax sp.
Budashevaella sp.
Ammobaculites sp.

Fig. S7
